# Chromatin Accessibility-Based Characterization of the Gene Regulatory Network Underlying *Plasmodium falciparum* Blood-Stage Development

**DOI:** 10.1016/j.chom.2018.03.007

**Published:** 2018-04-11

**Authors:** Christa Geeke Toenhake, Sabine Anne-Kristin Fraschka, Mahalingam Shanmugiah Vijayabaskar, David Robert Westhead, Simon Jan van Heeringen, Richárd Bártfai

**Affiliations:** 1 Radboud University, Faculty of Science, Department of Molecular Biology, Nijmegen, 6525 GA, the Netherlands; 2 Radboud University, Faculty of Science, Department of Molecular Developmental Biology, Nijmegen, 6525 GA, the Netherlands; 3 School of Molecular and Cellular Biology, Faculty of Biological Sciences, University of Leeds, Leeds LS2 9JT, UK

**Keywords:** malaria, *Plasmodium falciparum*, chromatin, ATAC-seq, RNA-seq, transcription factor, regulatory sequences

## Abstract

Underlying the development of malaria parasites within erythrocytes and the resulting pathogenicity is a hardwired program that secures proper timing of gene transcription and production of functionally relevant proteins. How stage-specific gene expression is orchestrated *in vivo* remains unclear. Here, using the assay for transposase accessible chromatin sequencing (ATAC-seq), we identified ∼4,000 regulatory regions in *P. falciparum* intraerythrocytic stages. The vast majority of these sites are located within 2 kb upstream of transcribed genes and their chromatin accessibility pattern correlates positively with abundance of the respective mRNA transcript. Importantly, these regions are sufficient to drive stage-specific reporter gene expression and DNA motifs enriched in stage-specific sets of regulatory regions interact with members of the *P. falciparum* AP2 transcription factor family. Collectively, this study provides initial insights into the *in vivo* gene regulatory network of *P. falciparum* intraerythrocytic stages and should serve as a valuable resource for future studies.

## Highlights

• Genome-wide mapping of regulatory DNA elements in *P. falciparum* blood stages by ATAC-seq
• Chromatin accessibility patterns predict transcriptional dynamics of parasite genes
• Accessible regions are sufficient to induce stage-specific gene expression
• Predicted *cis*-regulatory elements interact with ApiAP2 transcription factors

## Graphical Abstract

**Figure:**
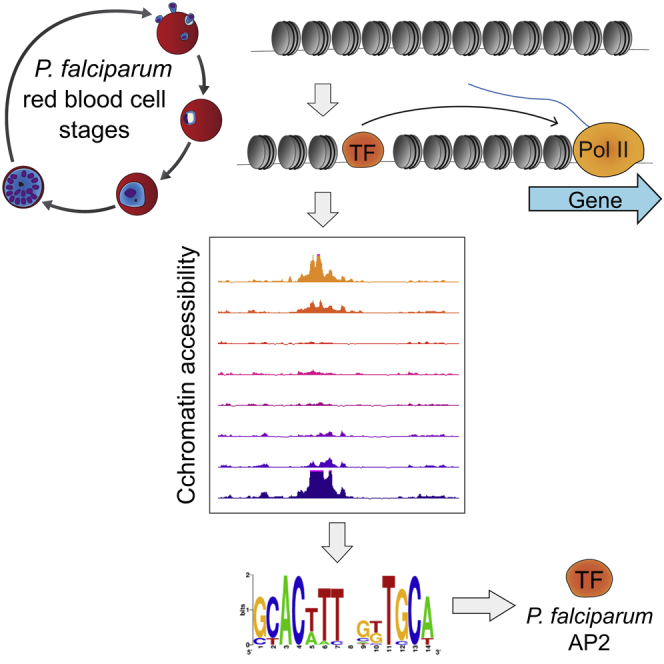

## Teaser

Proliferation of malaria parasites in human erythrocytes requires the expression of functionally related sets of proteins in a “just-in-time” manner. Toenhake et al. map the chromatin structure of blood-stage *P. falciparum* and use it to dissect the interplay between DNA elements and transcription factors controlling gene expression during intraerythrocytic development.

## Introduction

Malaria, caused by infection with parasites of the *Plasmodium* genus, remains a major health and economic burden (Murray et al., 2012). The parasite’s life cycle is intriguingly complex, requiring adaptation to several different host cell environments and transmission between the human host and the mosquito vector. The approximately 48 hr intraerythrocytic development of *P. falciparum* is responsible for most disease symptoms. It involves the invasion, remodeling, consumption, and rupture of human red blood cells while the parasite replicates by schizogony, giving rise to 16–32 new parasites (Cowman et al., 2016). Underlying this development and the pathogenicity of the parasite is a gene expression program that secures proper timing of gene transcription and production of functionally relevant proteins. However, despite being a fundamental eukaryotic process and a potential target of drug-based intervention, our understanding of gene expression regulation in *Plasmodium* is still in its infancy (Painter et al., 2011).

During the intraerythrocytic development cycle (IDC), the majority of genes are transcribed in a “just-in-time” manner, with peak mRNA abundances correlating with the need for the products they encode for (Bozdech et al., 2003). Although post-transcriptional and translational control mechanisms operate in this stage as well (Caro et al., 2014, Foth et al., 2011), the initial production of mRNAs, dictated by transcriptional and epigenetic mechanisms, remains a major and rate-limiting step in the gene expression process during the blood-stage cycle. In *P. falciparum*, epigenetic regulation of gene expression is most evident in heterochromatin-mediated gene silencing of, for example, antigenic variation genes, selection of erythrocyte invasion pathways, and control of gametocyte conversion rate (for review, see Voss et al., 2014). This type of regulation is, however, limited to genes located in subtelomeric regions and a few chromosome-internal heterochromatic islands (Flueck et al., 2009, Salcedo-Amaya et al., 2009), while the largest part of the parasite genome is in a transcriptionally permissive, euchromatic state.

These observations collectively point to an important role for transcriptional control mechanisms in stage-specific gene expression regulation, including the action of *trans*-acting transcription factors (TFs) that bind to specific DNA sequences and stimulate or inhibit the assembly and/or activity of the RNA polymerase II pre-initiation complex. Such sequence-specific TFs are, however, relatively low in numbers, constituting roughly 1% of all protein-coding genes (Balaji et al., 2005, Bischoff and Vaquero, 2010) compared to ∼3% in yeast or 6% in human. Despite general scarcity of sequence-specific TFs, the relevance of the Apicomplaxan AP2 family of TFs in *Plasmodium* has become evident over the past decade, mainly through the use of knockout or knockdown experiments (Flueck et al., 2010, Iwanaga et al., 2012, Kafsack et al., 2014, Kaneko et al., 2015, Modrzynska et al., 2017, Santos et al., 2017, Sinha et al., 2014, Yuda et al., 2009, Yuda et al., 2015, Zhang et al., 2017). While these functional genomic approaches have been very powerful to dissect the function of TFs outside of the IDC, they could only suggest the essentiality of AP2 factors during the IDC. Furthermore, rather little is known about DNA elements that act in concert with these specific TFs. Most of our current understanding of *cis*-regulatory DNA elements stems from deletion analyses of promoters (e.g., López-Estraño et al., 2007, Militello et al., 2004, Sunil et al., 2008), *in silico* DNA motif predictions (e.g., Elemento et al., 2007, Gunasekera et al., 2007, Russell et al., 2015, Young et al., 2008), and protein-binding microarray studies defining the *in vitro* sequence preference of recombinant AP2 domains (Campbell et al., 2010). Although these studies have certainly been valuable and some of the DNA motif predictions could indeed be confirmed by chromatin immunoprecipitation sequencing (ChIP-seq) experiments (Kaneko et al., 2015, Santos et al., 2017), we still lack an accurate, genome-wide overview of *cis*-regulatory DNA elements and their activity *in vivo*.

The binding of specific *trans*-factors to the DNA is associated with the eviction and/or destabilization of nucleosomes, thereby creating a more “accessible” chromatin environment. As a first attempt to explore open chromatin structures in *P. falciparum*, formaldehyde-assisted isolation of regulatory elements (FAIRE-seq) has been employed (Ponts et al., 2010). While this study reported increased accessibility at active promoter regions, the resolution of the data was not sufficient to improve the identification of regulatory elements. In a previous study, we applied MNase sequencing to profile the nucleosome landscape and provided proof of principle that the chromatin environment of a predicted regulatory element is depleted of nucleosomes and that this signature could be used to predict active regulatory elements (Kensche et al., 2016). As a completion of these efforts, here we set out to identify gene regulatory elements *in vivo* and on a genome-wide scale by directly profiling chromatin accessibility using the assay for transposase accessible chromatin sequencing (ATAC-seq; Buenrostro et al., 2013). We combined ATAC-seq and directional RNA sequencing (RNA-seq) on eight tightly synchronized *P. falciparum* IDC stages to profile gene regulatory events. Furthermore, we combined bioinformatics, biochemical, and reporter gene assays to characterize these *cis*-regulatory elements and their interactions with TFs. Collectively, this study represents a major step toward dissection of the transcriptional regulation network of this deadly pathogen and provides a valuable resource for future studies aiming to characterize or use gene regulatory elements.

## Results

### ATAC-Seq Identifies Accessible Chromatin Regions in the AT-Rich *Plasmodium* Genome

**Figure 1:**
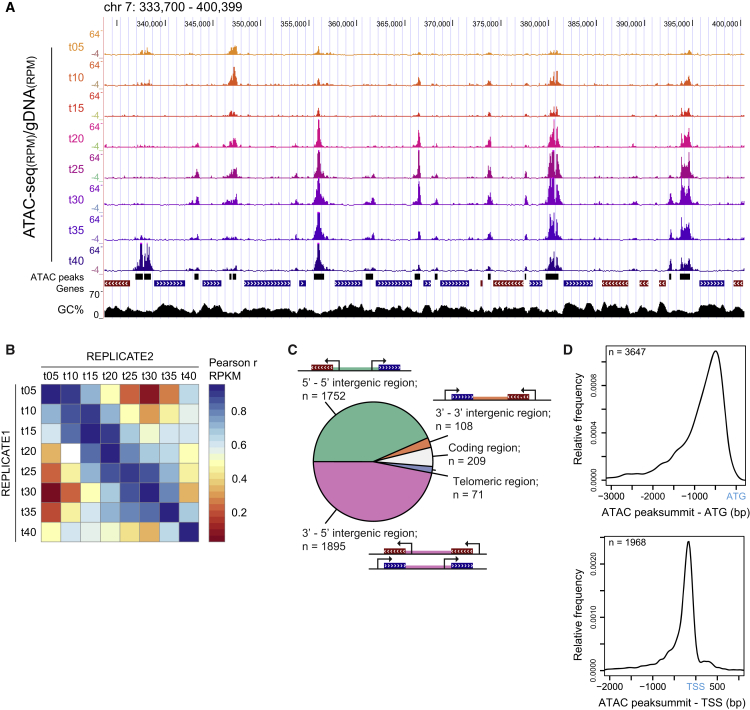
ATAC-Seq Detects Chromatin Accessibility during Intraerythrocytic Development of *P. falciparum* (A) UCSC genome browser screenshot of a 66,700 bp region on chromosome 7 displaying chromatin accessibility profiles from eight time points (t05–t40 hr post-invasion [hpi]) as a ratio of normalized ATAC-seq tag count over background transposition in naked, genomic DNA (ATAC-seq/gDNA). Black bars below t40 track depict the peak regions as identified by MACS2 peak calling. Coding sequences are indicted as blue (positive strand) or red (minus strand) bars. GC%, the mean percentage of GC nucleotides, smoothened over 5 bp windows. (B) Heatmap depicting the Pearson correlation of RPKM (reads per kb per million mapped reads) values in peak regions from the above dataset (REPLICATE1) and an independent eight time point ATAC-seq dataset (REPLICATE2) demonstrating a high degree of reproducibility. (C) The distribution of accessible regions among different genomic regions. Peaks were called on accessibility profiles of individual time points (t05–t40) and merged, yielding 4,035 accessible regions. Intergenic regions were categorized based on the direction of transcription of the flanking coding regions (divergent, green; convergent, orange; tandem, pink). (D) Peaks located in divergent and tandem intergenic regions were assigned to the closest downstream gene and the distance between the ATAC peak summit and the ATG or TSS was calculated. The line depicts the smoothened distribution of distances (kernel density estimate). See also Figure S1 and Table S1.

To identify and profile TF-binding events, we performed ATAC-seq on synchronized *P. falciparum* 3D7 parasites at eight consecutive time points during their IDC (from 5 to 40 hr post-invasion [hpi]). Considerable signal was detected in coding sequences (Figure S1A, purple track “t40 all”) and in subtelomeric regions of the genome (data not shown). We reasoned that this was likely due to the sequence bias of the enzyme (Goryshin et al., 1998), in combination with the distinctly higher GC content of these sequences as compared to the AT-rich intergenic regions (Gardner et al., 2002). To correct for such biases as well as biases introduced during library preparation and sequencing, we performed the same assay on naked, genomic DNA (gDNA). This control library also showed a distinctly higher read count in the GC-richer coding sequences (Figure S1A, bottom gray track “gDNA all”) and subtelomeric regions. Furthermore, in the chromatin context, Tn5 transposition is known to give rise to (sub-)nucleosomal fragments (<150 bp) as well as fragments corresponding to mono-, di-, and tri-nucleosomes as a result of transposition in the vicinity of TF-binding sites and in linker regions between nucleosomes, respectively (Buenrostro et al., 2013). We therefore reasoned that selecting reads with a size between 50 and 150 bp could increase the signal-to-noise ratio for the detection of TF-binding sites. Indeed, compared to the other insert sizes, a higher proportion of 50–100 bp and 100–150 bp fragments mapped to intergenic, putative regulatory regions (Figures S1A and S1B) and to binding sites of an AP2 TF (AP2-I; Santos et al., 2017; Figure S1B; next paragraph). Based on the above observations, we decided to use only fragments with a size between 50 and 150 bp and corrected the derived read counts with the read counts detected in the gDNA control library in all follow-up analyses (Figure 1A). Finally, the robustness of the data was assessed by preparing a replicate ATAC-seq dataset (replicate 2), which showed a high degree of correlation with the first dataset (Pearson correlation of 0.88 and higher; Figures 1B and S1C). Accordingly, our ATAC-seq approach enables robust and accurate identification of accessible chromatin regions despite AT richness of the *P. falciparum* genome.

### Dynamic Chromatin Accessibility in 5′ Intergenic Regions Highlights TF-Binding Events

**Figure 2:**
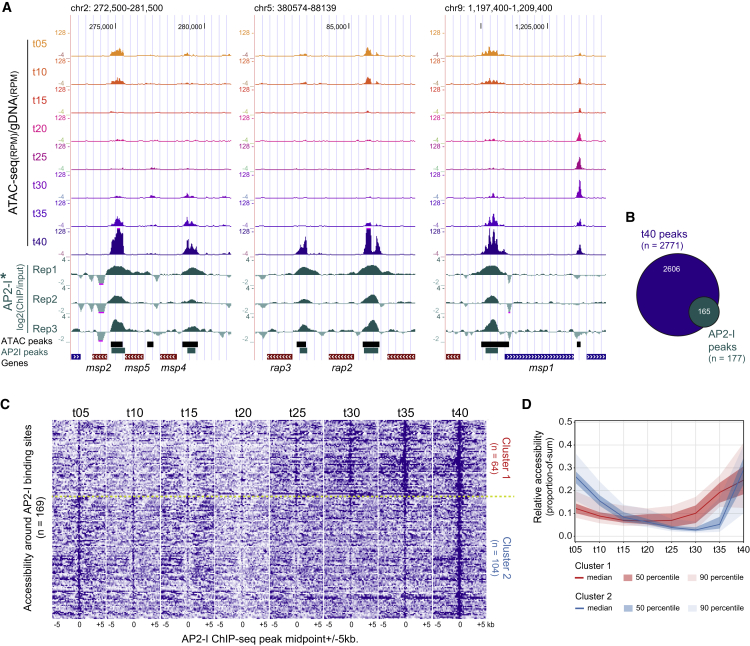
ATAC-Seq Predicts TF-Binding Events Detected Earlier by ChIP-Seq (A) UCSC genome browser screenshots of three genomic regions with AP2-I TF-binding sites. Normalized ATAC-seq coverage is plotted as ratio over coverage in gDNA control. Data of the three AP2-I ChIP-seq replicates from Santos et al. (2017), generated in the *P. falciparum* Dd2 line, were mapped against the *P. falciparum* 3D7 genome. Turquoise bars are AP2-I peaks from Santos et al. (2017). (B) Overlap between AP2-I peaks and ATAC peaks from t40 time point (165 out of 2,771 peaks overlap with 169 out of 177 AP2-I peaks) plotted as Venn diagram. (C) Heatmap of non-gDNA corrected ATAC-seq accessibility profiles over AP2-I peaks (midpoint ± 5 kb) that overlap with merged ATAC peaks (n = 169). Profiles were clustered using Pearson correlation calculated for the middle 100 bp window into 2 clusters by k-means clustering. (D) Median accessibility profiles for the two clusters of ATAC peaks defined in (C) with the 50^th^ and 90^th^ percentile as a dark- and light-colored shades. Accessibility is calculated as proportion of sum of quantile normalized RPKM values over the time points. See also Table S2.

Next, we identified local regions of increased accessibility for all eight time points of the IDC using the model-based analysis of ChIP-seq 2 (MACS2) algorithm for peak calling (Liu, 2016). The number of identified accessible regions reflects the overall transcriptional output at the given stage of development (Bártfai et al., 2010, Lu et al., 2017, Sims et al., 2009), with ∼500 regions in ring stages to ∼3,000 in late trophozoite/early schizonts (Figure S1D). After merging the peaks for all time points, a total 4,035 regions were identified that show increased accessibility during one or more stages of the IDC (Table S1), 92% of which were confirmed by the peaks called on the replicate ATAC-seq dataset (data not shown). Ninety percent of the accessible regions locate to intergenic regions containing one or two putative promoter regions (Figure 1C). Within these regions, the majority of peaks locate up to 2 kb upstream of the ATG and, when a transcription start site (TSS) is known (Kensche et al., 2016), within 1.5 kb upstream of the TSS (Figure 1D). In addition, these ATAC peaks captured 95% of the AP2-I-binding sites detected by ChIP-seq (Figures 2A and 2B; Santos et al., 2017). Interestingly, two different clusters of AP2-I-binding sites could be discriminated based on their accessibility profile during the IDC. A cluster of 64 regions (linked to 50 genes) becomes accessible in late trophozoites/early schizonts and a cluster of 105 regions (linked to 100 genes) becomes accessible in mature schizonts and shows increased accessibility in t05 rings (Figures 2C and 2D). This subdivision is also evident at the molecular level with genes in cluster 1 being enriched for processes related to chromatin organization and cell-cycle progression, while cluster 2 genes are clearly involved in host cell invasion (Table S2). Collectively, these results demonstrate that ATAC-seq detects dynamic chromatin accessibility in promoter regions of *P. falciparum* genes during the IDC and that it can capture TF-binding events. In addition, it demonstrates that data from ATAC-seq performed at multiple developmental stages can provide valuable temporal resolution to TF ChIP-seq data performed at a single time point.

### Chromatin Accessibility Patterns Are Predictive for Gene Expression Dynamics

**Figure 3:**
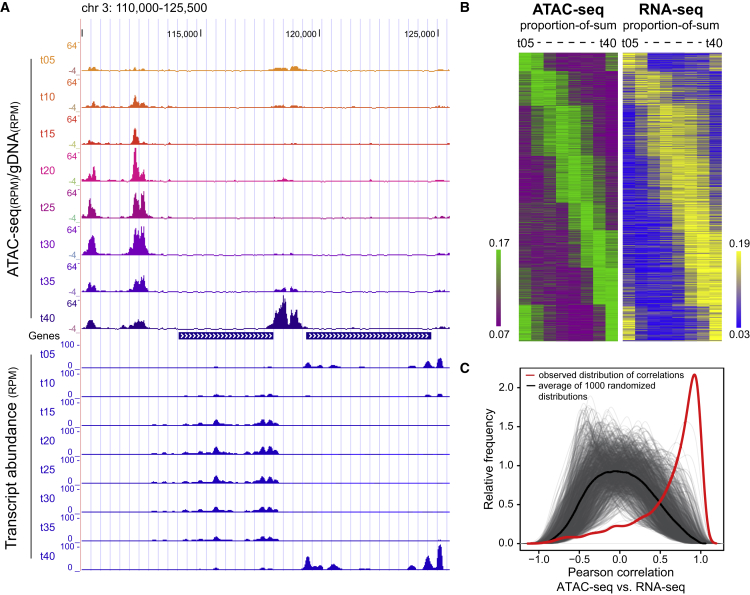
Chromatin Accessibility Positively Correlates with mRNA Abundance of the Downstream Gene (A) UCSC genome browser screenshot (chr3: 110,000–125,500) of ATAC-seq chromatin accessibility profiles (top) and corresponding RNA-seq profiles (bottom; only reads mapping to the sense strand are displayed). (B) Heatmaps displaying relative chromatin accessibility and mRNA abundance profiles of the downstream gene through eight stages of intraerythrocytic development. Relative accessibility in ATAC-seq peaks located in divergent or tandem intergenic regions was calculated as a proportion of sum of quantile normalized RPKM values over the time points and clustered by k-means using the 1-Pearson correlation distance metric. The relative transcript abundances of the downstream located genes, expressed as proportion of sum of sense strand RPKM values over the time points, were plotted in the same order. Color scales range from the 20^th^ to the 80^th^ percentile for both datasets. (C) Observed (red line) and 1,000 randomized distributions (1,000 gray lines with mean of all randomizations as black line) of Pearson correlation coefficients between relative chromatin accessibility and mRNA abundance of the downstream gene (as in B) plotted as smoothened kernel density distribution.

To assess the relationship between chromatin accessibility and gene expression, we prepared directional RNA-seq libraries from the same parasite cultures as used for ATAC-seq. Overall, the chromatin accessibility pattern and the transcript abundance pattern of the downstream gene are positively correlated (see examples in Figure 3A). To quantify this correlation, accessible regions were assigned to the closest downstream located gene, yielding 3,210 accessible region-gene pairs (accessible regions and/or genes with low signal and hence potentially noisy patterns were excluded; STAR Methods). Chromatin accessibility patterns during the IDC, which were highly reproducible between the two ATAC-seq replicates (Figure S1E; median correlation of r = 0.84), were then used to group accessible regions by means of k-means clustering. Alignment with the assigned genes revealed a high degree of similarity between chromatin accessibility patterns and relative abundance of corresponding mRNAs (Figure 3B). In fact, the majority of the genes showed a clear positive correlation between chromatin accessibility and relative mRNA abundance (Pearson correlation > 0.6; Figure 3C), demonstrating that chromatin accessibility is highly predictive of the gene expression pattern for the majority of genes. Moreover, this observation suggests that the gene regulatory events governing the IDC of *P. falciparum* are mainly activating events.

### ATAC-Seq Regions Are Sufficient for Regulating Stage-Specific Gene Expression

**Figure 4:**
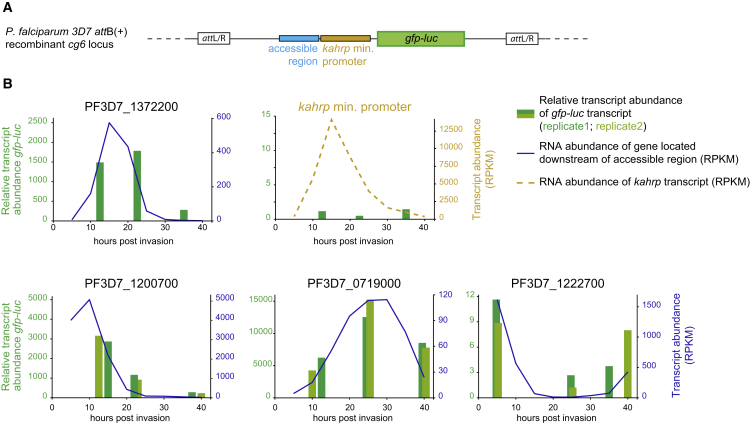
Accessible Regions Are Sufficient in Driving Stage-Specific Reporter Gene Expression (A) Schematic representation of the DNA constructs inserted into the *cg6* locus of *att*B(+) 3D7 *P. falciparum* parasites using the site-specific integration system (Nkrumah et al., 2006). Accessible regions detected by ATAC-seq (light blue) were cloned upstream of the minimal *kahrp* promoter (yellow) followed by the *gfp-luc* fusion gene (green). (B) Bar plots showing the relative *gfp-luc* transcript abundance determined by qRT-PCR for ring, trophozoite, and schizont stages from parasites carrying the reporter construct without accessible region (only the *kahrp* minimal promoter, yellow), with the accessible region of PF3D7_1372200 (*hrpIII*, positive control), or with selected accessible region located upstream of PF3D7_0719000, PF3D7_1200700, or PF3D7_1222700. For the latter three constructs with novel putative regulatory sequences, reporter gene expression was measured in biological duplicate. Relative *gfp-luc* transcript abundance was determined based on a standard reference dilution series (STAR Methods). The relative abundances in replicate 2 were scaled to the average of replicate 1. The raw data are depicted in Figure S2. Transcript abundance (RPKM) of the respective gene as determined by RNA-seq is indicated as a blue line for eight time points (t05–t40). See also Figure S2.

To study the potential of the identified accessible regions to drive stage-specific gene expression, parasite lines were generated with different accessible regions cloned upstream of the minimal *kahrp* promoter (Brancucci et al., 2012) and a *gfp-luciferase* (*gfp-luc)* reporter gene (Figures 4A, S2A, and S2B). The region upstream of PF3D7_1372200 (*hrpIII*) has been characterized before and functioned as a positive control (López-Estraño et al., 2007) while the accessible regions upstream of PF3D7_0719000, PF3D7_1200700, and PF3D7_1222700 were selected based on their stage-specific accessibility and RNA abundance profiles (Figure S2C, blue framed rectangle). In addition, for PF3D7_0719000 and PF3D7_1200700 we created control parasite lines with a neighboring, not-accessible intergenic region cloned upstream of the minimal *kahrp* promoter (Figure S2C, red framed rectangle; for PF3D7_1222700, integration of the negative control construct could not be achieved). Remarkably, for all tested accessible regions, the temporal expression profile of the reporter gene matched the RNA expression profiles of the respective downstream located genes and was clearly above the background detected in the control lines (Figures 4B and S2C). This demonstrates that intergenic regions displaying dynamic chromatin accessibility are sufficient to induce stage-specific expression of the downstream located gene.

### Specific Sequence Motifs Are Associated with Dynamics of Accessible Regions

**Figure 5:**
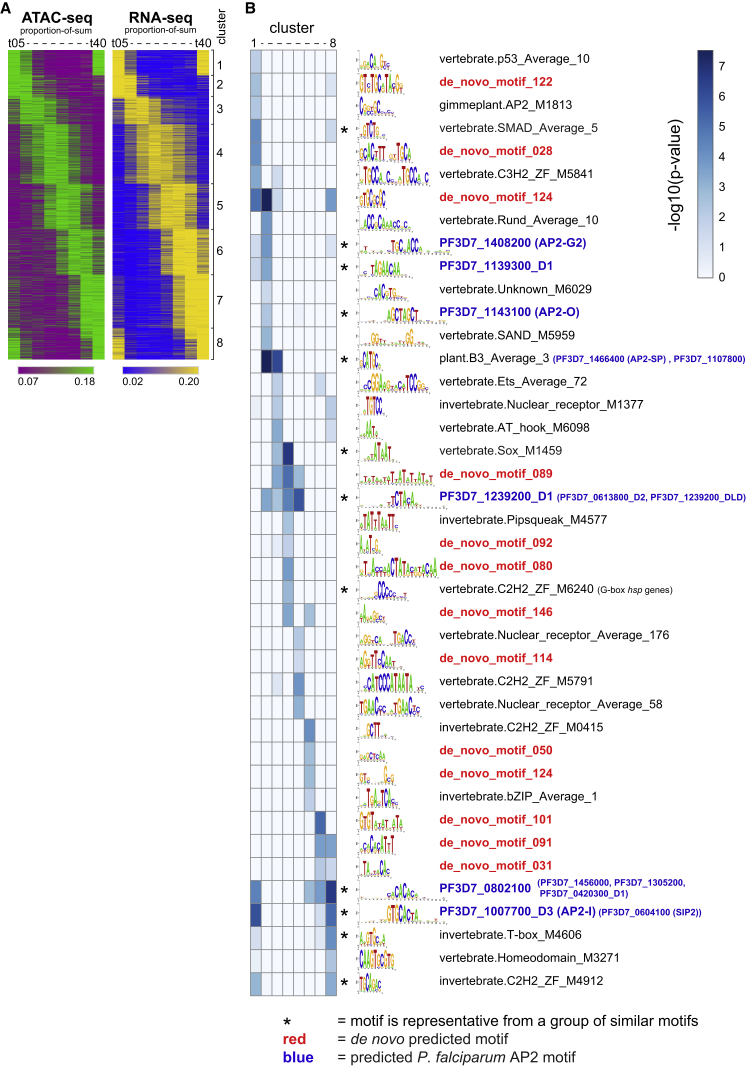
DNA Motifs Enriched in Stage-Specific ATAC Regions (A) Co-clustering of ATAC-seq and RNA-seq data, limited to peak-to-gene pairs with Pearson correlation > 0.6 (n = 2,118; Table S1). Accessibility and transcript abundance are expressed as proportion of sum of (qn)RPKM values over the time points and clustered by k-means using the 1-Pearson correlation distance metric into eight clusters. Color scales range from the 20^th^ to 80^th^ percentile per dataset. (B) Heatmap of significance estimates for differential motif enrichment (expressed as −log10(p value)). Each column relates to a cluster generated by co-clustering the ATAC-seq and RNA-seq data by k-means cluster (see A). Each row refers to a motif or a “motif group,” with logo and name listed on the right. Asterisks indicate that the motif is a representative from a group of similar motifs (Figure S3B; Table S3). Predicted binding sites for *P. falciparum* AP2 TFs are reported in blue font with PlasmoDB geneID (name, if known, is in brackets). When a cluster contains one or more predicted *P. falciparum* AP2 motifs, these are reported in brackets behind the representative motif. See also Figure S3 and Tables S1 and S3.

The ATAC-seq data revealed different patterns of accessibility over the IDC that showed an overall positive correlation with mRNA abundance. We reasoned that these were likely caused by the presence of different DNA motifs in promoter regions that are bound by specific TFs in a stage-specific manner. To identify DNA motifs that could perform this function, we first performed an exhaustive *de novo* motif search using GimmeMotifs and seqGL (STAR Methods; Setty and Leslie, 2015, van Heeringen and Veenstra, 2011). These *de novo* predicted motifs were combined with previously predicted *Plasmodium* motifs (Campbell et al., 2010) and known vertebrate, invertebrate, and plant motifs from the CIS-BP database (Weirauch et al., 2014), yielding a comprehensive library of putative *cis*-regulatory sequences. Next we identified gene sets with clear stage-specific accessibility/expression profiles by selecting all accessible regions that positively correlated with transcript abundance (Pearson correlation > 0.6; n = 2,118 regions; Table S1) and clustered those considering both their accessibility and transcript abundance patterns over the IDC into eight clusters using k-means clustering (Figure 5A).

To identify motifs associated with specific accessibility/expression patterns, we used an ensemble of different regression and classification methods, as implemented in GimmeMotifs (van Heeringen and Veenstra, 2011), and searched for motifs from the above library that were consistently enriched in accessible regions of a specific cluster (p < 0.01, in at least two out of three runs; Table S3). After manually removing eight low-information content motifs (Figure S3A; Table S3), we clustered the remaining motifs, yielding 41 non-redundant motifs (Figures 5B and S3B; Table S3; for redundancy filtering, see van Heeringen and Veenstra, 2011). Interestingly, for all ATAC/RNA-seq co-clusters we observed enrichment of at least one predicted AP2 motif (in total, 16 motifs predicted for 13 different AP2 proteins; blue font in Figure 5B), suggesting that the corresponding AP2 TF is likely relevant in regulating these genes. Additionally, we detected motifs similar to the G-box element upstream of heat shock genes (motif vertebrate.C2H2_ZF_M6240; Militello et al., 2004). Importantly, in addition to these previously predicted motifs, we identified 13 *de novo* motifs with potential regulatory capacity in *P. falciparum* (indicated with red font in Figure 5B).

### DNA Pull-Down Combined with Quantitative Proteomics Reveals *cis*-*trans* Regulatory Interactions

We selected four motifs and identified their protein interactors by performing DNA pull-downs using short oligos representing actual accessible sequences containing the selected motifs and native nuclear extracts from non-synchronous, asexual *P. falciparum* 3D7 cultures. To identify proteins that specifically bind to the motif, but not to a control oligo with a scrambled motif, we analyzed pull-down and control samples by quantitative tandem mass spectrometry. (See Table S4 for the complete list of motifs and identified proteins.)

**Figure 6:**
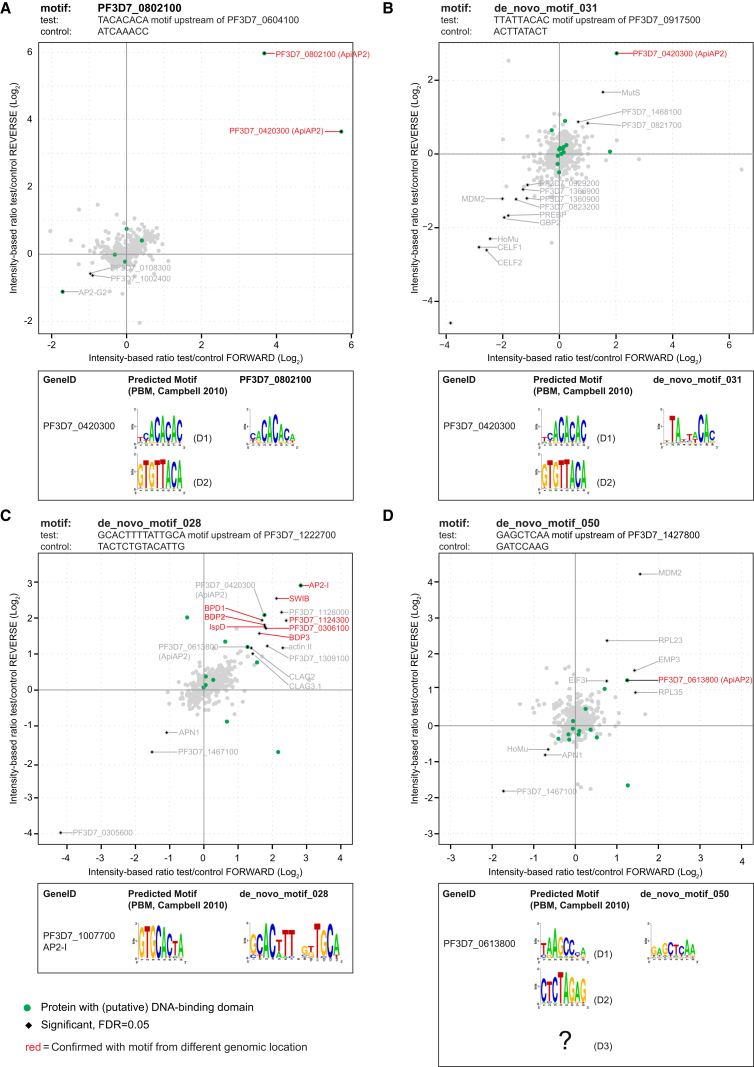
DNA Pull-Downs Identify Potential *cis*-*trans* Regulatory Interactors Scatterplots displaying the quantitative proteomic analysis of duplicate DNA pull-downs with label swap using (A) 58–60 bp DNA probes with the CA-repetitive motif (ACACACAT) and three *de novo* predicted motifs (B, de_novo_motif_031, TTATTACAC; C, de_novo_motif_028, GCACWWTNNKTGCW; and D, de_novo_motif_050, GAGCTCAA). The same probes with a scrambled motif were used as controls. The statistically significant outliers (black diamond, intensity-based FDR < 5%) are the potential interactors to the motif. Red font indicates that the interaction was confirmed using a probe from a different genomic region, but containing the same motif. Green dots are candidate DNA-binding factors derived from Table 4 of Bischoff and Vaquero (2010). Earlier predicted binding sites for the identified AP2 factors are show in relation to the motif used in the pull-down below each plot. See also Figure S4 and Table S4.

First, we tested the CA-repeat motif predicted for protein PF3D7_0802100, which formed a “motif group” with similar motifs predicted for other AP2 proteins (PF3D7_0420300, PF3D7_1305200, and PF3D7_1456000; Figure S3B). The DNA pull-down confirmed the specific recruitment of PF3D7_0802100 and PF3D7_0420300 to the ACACACAT motif when compared to a scrambled control motif (ATCAAACC), but not the other two factors (Figure 6A).

Next, we tested three *de novo* motifs (031, 028, and 050). All these motifs captured at least one AP2 TF (Figures 6B–6D). The AP2 factor PF3D7_0420300 was consistently identified among the interactors of the TTATTACAC motif (de_novo_motif_031; Figures 6B and S4A). Remarkably, this motif is more similar to the sequence preference of the second AP2 domain of this factor (TTATTACAC versus GTGTTACA; Campbell et al., 2010), potentially suggesting that this factor can bind to two different regulatory elements (CACACACA, see above, and TRTTACAC) using different AP2 domains.

For the second *de novo* motif (028), we tested three different probes, and interestingly, partially overlapping sets of chromatin-related factors were enriched together with the TF AP2-I in each of them (Figures 6C, S4B, and S4C). These included bromodomain proteins (BDP) 1, 2, and 3 (PF3D7_1475600); HMGB3; and an SWIB/MDM2 domain-containing protein (SWIB, PF3D7_0611400), but also two conserved unknowns (PF3D7_0306100 and PF3D7_1124300), NOP5 and IspD. This suggests that AP2-I is sufficient to recruit these chromatin factors to target gene promoters, in line with the current study of Santos et al. (2017). Notably, in two of the three pull-downs another AP2-factor, SIP2, was enriched with the motif-containing oligos (Figures S4B and S4C). SIP2 was first predicted to bind to a motif very similar to AP2-I (Figures S4B and S4C; Campbell et al., 2010) but was later shown to a bind a longer sequence named SPE2 (NVTGCA-4(5)-VGTGCR) upstream of subtelomeric *var* genes (Flueck et al., 2010). By chance, sequences similar to a full SPE2 motif can be found in both of these oligos, including some flanking sequences (Table S5), explaining the binding of SIP2 to these two, but not the first oligo. Hence, SIP2 is likely not a genuine interactor of de_novo_motif_028.

Lastly, we tested de_novo_motif_050 (GAGCTCAA) using DNA probes from two different genomic regions. In this case, we observed a moderate, but consistent, interaction with the AP2 factor PF3D7_0613800 (Figures 6D and S4D). This motif is different from the predicted binding sites of domains 1 and 2 of PF3D7_0613800 (Campbell et al., 2010) and might be recognized by its third domain for which sequence preference has not been defined.

Collectively, these experiments indicate that the motifs predicted from the stage-specific regulatory elements indeed interact with specific TFs. In addition, they point to a delicate interplay between DNA elements, and transcription and chromatin-modifying factors in regulating intraerythrocytic development of malaria parasites.

## Discussion

Here we present high-resolution temporal chromatin accessibility data during intraerythrocytic development of *P. falciparum*. ATAC-seq, used in this study (Figure 1), clearly supersedes other chromatin-based technologies such as FAIRE-seq (Harris et al., 2011, Ponts et al., 2010), MNase-seq (Kensche et al., 2016), or ChIP-seq (Ubhe et al., 2017) in identifying active regulatory sites on a global scale, both in terms of the number of sites identified and in terms of spatial resolution of the data. Furthermore, ATAC-seq shows a nearly complete overlap with ChIP-seq profiles of a TF, AP2-I (Figure 2; Santos et al., 2017). While due to the bias of the transposase against AT-rich sequences and stringent filtering we might not detect all regulatory events, we identified an accessible region for at least 60% of all *P. falciparum* genes and about 70% of the genes expressed during the IDC (comprising about 85% of all genes; Otto et al., 2010), providing a valuable resource for future studies that could range from targeted gene studies to predicting functional consequences for SNPs.

Notably, the coupling between accessible regions and genes in our analysis was based on the assumption that most genes in the *P. falciparum* genome are regulated by the nearest regulatory elements. While this assumption might not always be correct, it is remarkable that temporal accessibility and mRNA abundance profiles strongly correlate for the majority of the genes (Figure 3), suggesting functional interactions between these regulatory elements and the nearby genes. Furthermore, the DNA sequence of all four tested accessible regions was sufficient in dictating a stage-specific expression pattern to a reporter gene that is similar to that of the respective endogenous gene (Figure 4). Although our data do not exclude the existence of distant enhancers, at least the majority of identified regions in this dataset likely operate at close distance to their target gene. The scarcity of distant regulatory sequences in *P. falciparum* is also supported by the fact that long-distance interactions identified so far in chromosome conformation studies in this parasite were restricted to centromeres, ribosomal DNA loci, and subtelomeric regions (Ay et al., 2014, Lemieux et al., 2013). Collectively, our data, together with earlier studies (e.g., Hasenkamp et al., 2013, Kaneko et al., 2015, López-Estraño et al., 2007, Santos et al., 2017, Ubhe et al., 2017, Yuda et al., 2015), suggest that most *P. falciparum* genes have a compact regulatory unit like other unicellular eukaryotes (i.e., yeast), with minimal promoter(s) and upstream regulatory element(s) located within 1 or 2 kb of the target gene. However, our data, in combination with future high-resolution chromosome conformation studies, might reveal distant enhancers, if they exist.

The marked positive correlation between chromatin accessibility and mRNA abundance (Figure 3) also supports the notion that activating, rather than repressive, regulatory events drive gene expression in the IDC of *P. falciparum*. Alternatively, ATAC-seq might preferentially detect activator bound chromatin regions. Yet ATAC-seq has been shown to detect non-transcription-related DNA-binding events (e.g., CTCF binding to insulator elements; Buenrostro et al., 2013) and bivalent promoters (co-occurrence of activating and repressive histone markings) in other organisms (Minoux et al., 2017, Xu et al., 2017). Also, thus far, only two TFs, AP2-G2 and AP2-SP, have been postulated to have repressive properties during blood-stage development of *P. berghei* (Modrzynska et al., 2017, Sinha et al., 2014, Yuda et al., 2015). Regardless of the presence and specific functions of a few transcriptional repressors, our data suggest that a cascade of transcription-activating events is mainly responsible for the stage-specific expression during blood-stage development of *P. falciparum*.

Since their discovery, the ApiAP2 gene family has been regarded as the major family of putative TFs in *Plasmodium*. However, even with these 27 putative TFs, the proportion of TFs to the total number of genes remains low (∼50–60 among ∼5,800 genes compared to, for example, 169 per ∼6,000 genes in yeast; Hahn and Young, 2011). Therefore, to our surprise, besides AP2s, we did not consistently detect any other protein family in our DNA pull-downs that could function as a sequence-specific DNA-binding factor. Hence, despite the existence of few other types of DNA-binding factors in *Plasmodium* (including C2H2-type [Bertschi et al., 2017], Myb-type [Gissot et al., 2005], and HMGB-domain proteins [Briquet et al., 2006]), so far all evidence suggests that the ApiAP2 family can be regarded as the major TF family in *Plasmodium*, leaving researchers puzzled as to how such a small number of factors can govern such a delicate gene expression program. Combinatorial action of multiple TFs has been suggested to increase the regulatory potential of these factors in directing development of malaria parasites (e.g., Russell et al., 2015, van Noort and Huynen, 2006). Such cooperative interaction between AP2-I and other stage-specific TFs could explain the different accessibility patterns observed for the AP2-I-binding sites (Figure 2D). Yet we did not find any DNA motifs, other than the AP2-I-binding site (GTGCA), strongly enriched in these clusters that could serve as a binding site for such factor (data not shown). Alternatively, post-translational modifications of DNA-binding domains from TFs (Cobbold et al., 2016) or protein-protein interactions between TFs and cofactors could affect TF sequence specificities and/or recruitment of TFs to specific chromatin regions (Levo and Segal, 2014). To this end, we (Figure 6C) and others (Josling et al., 2015, Santos et al., 2017) detected a strong interaction between AP2-I and an epigenetic complex involving, among others, two acetylated histone-binding proteins (BDP1 and BDP2). However, if and how these proteins contribute to stage- and/or sequence-specific binding patterns of AP2-I or enhanced binding of AP2-I to acetylated chromatin regions remains to be determined. Furthermore, nearly half of the AP2 TFs have more than one AP2 domain. Our pull-down data suggest that in fact some of the AP2 factors could interact with different regulator elements using different domains (Figure 6). Eventually, it seems conceivable that the limited number of sequence-specific TFs encoded by the *Plasmodium* genome use the combination of the above mechanisms to achieve the precision of regulation required to drive the gene expression program underlying blood-stage development. Collectively, our work provides the in-depth global view of the *in vivo* transcriptional regulatory events during intraerythrocytic development of *P. falciparum*. It also highlights some intricate details of the interplay between TFs and *cis*-regulatory elements that controls gene transcription, bringing us a big step closer to understanding and fighting this deadly parasite.

## STAR★Methods

### Key Resources Table

**Table:** 

REAGENT or RESOURCE	SOURCE	IDENTIFIER
**Bacterial and Virus Strains**		

STBL3_pDC2	(Nkrumah et al., 2006)	N/A
STBL3_pOM1	This manuscript	N/A
STBL3_pOM2	This manuscript	N/A
XL10-Gold_*att*P_min*kahrp*	This manuscript	N/A
XL10-Gold_*attP_*min*kahrp_PF3D7_1372200*	This manuscript	N/A
XL10-Gold_*attP_*min*kahrp_PF3D7_1200700*	This manuscript	N/A
XL10-Gold_*attP_*min*kahrp_PF3D7_0719000*	This manuscript	N/A
XL10-Gold_*attP_*min*kahrp_PF3D7_1222700*	This manuscript	N/A
XL10-Gold_*attP_*min*kahrp_PF3D7_1200700*negative	This manuscript	N/A
XL10-Gold_*attP_*min*kahrp_PF3D7_0719000*negative	This manuscript	N/A
DH5α_pINT	This manuscript	N/A
STBL3	Thermo Fisher Scienfic	Cat#C7373-03
XL10-Gold	Stratagene	Cat#200314
DH5α	New England Biolabs	Cat#C29871

**Chemicals, Peptides, and Recombinant Proteins**		

Blasticidin-S-HCl	Thermo Fisher Scienfic	Cat#R210-01
WR99210	Jacobus Pharmaceuticals	N/A
Geneticin	Thermo Fisher Scienfic	Cat#11811-031
Proteinase K	Sigma-Aldrich	Cat#P6556
KAPA HiFi HotStart ReadyMix	KAPA Biosystems	Cat#KK2602
TURBO DNase	Ambion (Thermo Fisher Scientific)	Cat#AM2238
NextFlex adapters	Bio Scientific	Cat#514122
Actinomycin D	Thermo Fisher Scientific	Cat#11805017
SuperScript III Reverse Transcriptase	Invitrogen (Thermo Fisher Scientific)	Cat#18080044
RNasin Plus RNase Inhibitor	Promega	Cat#N261B
2x iQ SYBR Green Supermix	BioRad	Cat#170-8887
cOmplete, EDTA-free Protease Inhibitor Cocktail	Roche (Sigma-Aldrich)	Cat#04693132001
Ribonucleic acid, transfer from baker’s yeast (*S. cerevisiae*)	Sigma-Aldrich	Cat#R5636
Poly(deoxyinosinic-deoxycytidylic) acid sodium salt	Sigma-Aldrich	Cat#P4929
Poly(deoxyadenylic-thymidylic) acid sodium salt	Sigma-Aldrich	Cat#P0883
TCEP	Sigma-Aldrich	Cat#C4706-2G
MMTS	Thermo Fisher Scienfic	Cat#23011
Trypsin/Lys-C Mix, Mass Spec Grade	Promega	Cat#V5072
NaBH_3_CN	Merck	Cat#818053
NaBD_3_CN	Sigma-Aldrich	Cat#190020-1G
Trifluoroacetic acid ULC/MS	Biosolve BV	Cat#20234131

**Critical Commercial Assays**		

Wizard Plus SV Minipreps DNA Purification Systems	Promega	Cat#A1460
QIAamp DNA Blood Mini Kit	QIAGEN	Cat#51106
QIAquick PCR Purification Kit	QIAGEN	Cat#28106
MinElute PCR Purification Kit	QIAGEN	Cat#28006
QIAGEN RNeasy Mini Kit	QIAGEN	Cat#74106
Oligotex mRNA Mini Kit	QIAGEN	Cat#70022
Qubit dsDNA HS Assay Kit	Thermo Fisher Scientific	Cat#Q32854
Qubit RNA HS Assay Kit	Thermo Fisher Scientific	Cat#Q32852
Qubit Protein Assay Kit	Thermo Fisher Scientific	Cat#Q33212
Nextera DNA Library Prep Kit	Illumina	Cat#FC-121-1030
Nextera DNA Sample Preparation Index Kit	Illumina	Cat#FC-121-1012
Agilent High Sensitivity DNA Kit	Agilent	Cat#5067-4626
KAPA Library Quantification Kit	KAPA Biosystems	Cat#KR0405
NextSeq500/550 HighOutput kit V2 (75 cycles)	Illumina	Cat# FC-404-2005.

**Deposited Data**		

ATAC-seq data in *P. falciparum* 3D7	This manuscript	GEO: GSE104075
RNA-seq data in *P. falciparum* 3D7	This manuscript	GEO: GSE104075
AP2-I-GFP ChIP-seq data in *P. falciparum* Dd2	(Santos et al., 2017)	GEO: GSE80293
*P. falciparum* 3D7 reference genome (release 26)	PlasmoDB and GeneDB (Aurrecoechea et al., 2009, Logan-Klumpler et al., 2012)	http://plasmodb.org/common/downloads/release-26/Pfalciparum3D7/fasta/data/PlasmoDB-26_Pfalciparum3D7_Genome.fasta
*P. falciparum* 3D7 reference annotated transcriptome (realease 26)	PlasmoDB and GeneDB (Aurrecoechea et al., 2009, Logan-Klumpler et al., 2012)	http://plasmodb.org/common/downloads/release-26/Pfalciparum3D7/fasta/data/PlasmoDB-26_Pfalciparum3D7_AnnotatedTranscripts.fasta
*P. falciparum* 3D7 annotated proteome (release 9.3)	PlasmoDB and GeneDB (Aurrecoechea et al., 2009, Logan-Klumpler et al., 2012)	http://plasmodb.org/common/downloads/release-9.3/Pfalciparum3D7/fasta/data/PlasmoDB-9.3_Pfalciparum3D7_AnnotatedProteins.fasta
Motifs from plants, vertebrates and invertebrates reported in CISBP	(Weirauch et al., 2014)	http://cisbp.ccbr.utoronto.ca/index.php

**Experimental Models: Cell Lines**		

Parasite strain: *P. falciparum* 3D7	(Walliker et al., 1987)	Alan Cowman, WEHI, Melbourne, Australia
Parasite strain: *P. falciparum* 3D7 *att*B(+)	(Nkrumah et al., 2006)	David A. Fidock, Columbia Uni., US
Parasite strain: *P. falciparum* 3D7 *att*B::*att*P_min*kahrp*	This manuscript	N/A
Parasite strain: *P. falciparum* 3D7 *att*B::*att*P_min*kahrp*_PF3D7_137220	This manuscript	N/A
Parasite strain: *P. falciparum* 3D7 *att*B::*att*P_min*kahrp*_PF3D7_1200700	This manuscript	N/A
Parasite strain: *P. falciparum* 3D7 *att*B::*att*P_min*kahrp*_PF3D7_0719000	This manuscript	N/A
Parasite strain: *P. falciparum* 3D7 *att*B::*att*P_min*kahrp*_PF3D7_1200700negative	This manuscript	N/A
Parasite strain: *P. falciparum* 3D7 *att*B::*att*P_min*kahrp*_PF3D7_0719000negative	This manuscript	N/A

**Oligonucleotides**		

See Table S1 for primers used for cloning and RTqPCR	Biolegio B.V.	N/A
See Table S1 for DNA oligo’s used for DNA pull-down experiments	Integrated DNA Technologies	N/A
Random hexamer primers	Roche (Sigma-Aldrich)	Cat#11034731001
OligodT_12-18_	Invitrogen (Thermo Fisher Scientific)	Cat#18418012

**Recombinant DNA**		

MV163 plasmid	(Vos et al., 2015)	Robert Sauerwein, Radboud UMC, NL
pDC2 (*att*B containing plasmid)	(Nkrumah et al., 2006)	David A. Fidock, Columbia Uni., US
pINT	(Nkrumah et al., 2006)	David A. Fidock, Columbia Uni., US
pOM1	This manuscript	N/A
pOM2	This manuscript	N/A
*att*P_min*kahrp*	This manuscript	N/A
*att*P_min*kahrp_PF3D7_0719000*	This manuscript	N/A
*att*P_min*kahrp_PF3D7_1200700*	This manuscript	N/A
*att*P_min*kahrp_PF3D7_1222700*	This manuscript	N/A
*att*P_min*kahrp_PF3D7_1372200*	This manuscript	N/A
*att*P_min*kahrp_PF3D7_0719000*negative	This manuscript	N/A
*att*P_min*kahrp_PF3D7_1200700*negative	This manuscript	N/A
*att*P_min*kahrp_PF3D7_1222700*negative	This manuscript	N/A

**Software and Algorithms**		

FastQC v0.11.2	(Andrew, 2010)	RRID: SCR_014583; http://www.bioinformatics.babraham.ac.uk/projects/fastqc/
BWA samse (version 0.7.12-r1039)	(Li and Durbin, 2010)	RRID: SCR_010910; http://bio-bwa.sourceforge.net/bwa.shtml
BWA-mem (version 0.7.10)	(Li, 2013)	RRID: SCR_010910; http://bio-bwa.sourceforge.net/bwa.shtml
Picard tools (version 1.139)	Broad Institute	RRID: SCR_006525; https://broadinstitute.github.io/picard/
Samtools (version 1.2 and 1.3.1)	(Li et al., 2009)	RRID: SCR_002105; http://samtools.sourceforge.net/
Bedtools suite (version 2.20.1)	(Quinlan and Hall, 2010)	RRID: SCR_006646; http://bedtools.readthedocs.io/en/latest/
MACS2 (release 2.7)	(Liu, 2016)	RRID: SCR_013291; https://github.com/taoliu/MACS/wiki/Advanced:-Call-peaks-using-MACS2-subcommands
R package preprocessCore (version 1.36.0)	(Bolstad, 2017)	https://github.com/bmbolstad/preprocessCore
UCSC Genome Browser	(Kent et al., 2002)	RRID: SCR_005780; http://genome.ucsc.edu/
Morpheus tool	Broad Institute	https://software.broadinstitute.org/morpheus/
Trimmomatic (version 0.36)	(Bolger et al., 2014)	RRID: SCR_011848; http://www.usadellab.org/cms/?page=trimmomatic
Fluff	(Georgiou and van Heeringen, 2016)	https://github.com/simonvh/fluff/blob/master/README.md
maxQuant (version 1.5.3.30)	(Cox and Mann, 2008)	RRID: SCR_014485; http://www.coxdocs.org/doku.php?id=maxquant:start
Perseus software package (version 1.4.0.20)	(Tyanova et al., 2016)	RRID: SCR_015753; http://www.coxdocs.org/doku.php?id=perseus:start
R package SeqGL (version 1.1.3)	(Setty and Leslie, 2015)	https://bitbucket.org/leslielab/seqgl/overview
GimmeMotifs package (v0.11.0)	(van Heeringen and Veenstra, 2011)	RRID: SCR_001146; http://gimmemotifs.readthedocs.io/en/master/

**Other**		

Plasmodipur filters	EuroProxima	Cat#8011Filter25u
Agencourt AMPure XP beads	Beckman Coulter	Cat#A63882
Streptavidin Sepharose High Performance	GE Healthcare	Cat#17511301
2% E-Gel Size Select agarose gels	Invitrogen (Thermo Fisher Scientific)	Cat#G6610-02

### Contact for Reagent and Resource Sharing

Requests for resources and reagents should be directed to the Lead Contact, Richárd Bártfai (r.bartfai@science.ru.nl).

### Experimental Model and Subject Details

#### Parasite Culture Conditions

Parasites were cultured in RPMI medium supplemented with 10% human serum, 0.2% NaHCO_3_ and 2.5% or 5% human O+ red blood cells. Parasite lines were maintained in a shaking semi-automated 37c°C incubator in 10ml total volume and 5% hematocrit. For the ATAC-seq and RNA-seq parasite collections, the cultures were kept in T75 culture flasks with 20ml total volume and 2.5% or 1.25% hematocrit. For these collections the T75 flasks were placed in candle jars in a steady 37°C incubator, as in Kensche et al. (2016). For the collections of parasite RNA for RT-qPCR and parasite nuclei for the generation of nuclear protein extract, 20 or 50 mL parasite cultures with 2.5% hematocrit were kept in T75 or T175 flasks in a steady 37°C incubator with gas composition of 3% O_2_, 4% CO_2_ and 93% N_2_.

#### Parasite and Bacterial Strains

See Table S5 for details on parasite and bacterial strains used in this study.

### Method Details

#### Parasite Culture Synchronizations and Collections

For combined ATAC-seq and RNA-seq collections, cultures were selected for *var2csa* expression, expanded and synchronized as follows. VAR2CSA panning was performed as in Fraschka et al. (2016). Petri dishes (150 × 15 mm, BD biosciences Falcon 351058) were coated overnight with Chondroitin sulfate A (0.05% CSA in PBS) and blocked with 1% Casein/PBS solution for at least one hour and rinsed twice with RPMI. Parasite cultures were centrifuged, resuspended in RPMI with 10% human serum, transferred to the CSA-coated Petri dishes and incubated for 30 min at 37°C in a candle jar. Afterward, unbound parasites and non-infected erythrocytes were removed by gentle RPMI washes. Bound parasites were extensively resuspended in complete medium to detach them from CSA. Fresh blood was added to these parasites and they were put back in culture medium as described above in the shaking incubator. This selection was repeated four times before expansion. Before and during expansion of the culture, parasites were synchronized by sorbitol treatment and a Percoll gradient centrifugation. For the sorbitol treatment, parasites were spun down and the parasite pellet was gently resuspended in 6-7 pellet volumes of 5% D(-)-sorbitol (Merck, #107758) and incubated for 10 min at 37°C while shaking. Parasites were spun down and new medium and fresh blood were added to 5% hematocrit. For percoll gradients, parasite cultures were spun down, resuspended in fresh medium to 10% hematocrit and an equal volume of 63% Percoll (GE Healthcare, #17-0891-01) in PBS was gently layered below the culture. The schizont interface was collected after spinning the gradient and fresh, Plasmodipur filtered RBCs (EuroProxima, the Netherlands) were added a 1.5 h later which was then set as time point zero (0 hours post invasion (hpi)) resulting in a synchronicity window of 7 h (i.e., 7h difference between the first and last invasion). Medium was changed every ten hours but not less than ten hours before collection. Cultures were mixed with every medium change and after 20 hpi kept at 1.25% hematocrit. Parasites were collected from 5 hpi onward every 5 hours and ATAC-seq and RNA-seq collections were performed from the same synchronized culture. Giemsa stained blood smears were made at each time point to monitor parasite growth and staging (See Figure S5 for representative microscope images and Table S6 with counts of parasite stages per time point).

For collections of parasites carrying the *att*P*(+)_*min*kahrp* expression constructs, site-specific integration was first confirmed and parasites were synchronized using sorbitol treatments and Percoll gradient centrifugations as described above. For each parasite line ring, trophozoite and schizont stages were collected (PF3D7_0719000 replicate 1 synchronized to a ∼8 h window, collected 12 hpi, 25 hpi, 39 hpi; PF3D7_0719000 replicate 2 synchronized to a ∼10 h window, collected 10.3 hpi, 25 hpi, 40.5 hpi; PF3D7_1200700 replicate 1 synchronized to a ∼12 h window, collected 14 hpi, 23 hpi, 38 hpi; PF3D7_1200700 replicate 2 synchronized to a ∼10 h window, collected 12.25 hpi, 23 hpi, 39.5 hpi; PF3D7_1222700 replicate 1 synchronized to a ∼5 h window, collected 5 hpi, 25 hpi, 35 hpi; PF3D7_1222700 replicate 2 synchronized to a ∼8 h window, collected 6.25 hpi, 24.5 hpi, 41 hpi; PF3D7_1372200 synchronized to a ∼12 h window, collected 12 hpi, 23 hpi, 36 hpi; *kharp*minimal promoter only synchronized to a ∼5 h window, collected 5 hpi, 25 hpi, 35 hpi; PF3D7_0719000negative replicate 1 synchronized to a ∼5 h window, collected 8 hpi, 25 hpi, 38.75 hpi; PF3D7_0719000negative replicate 2 synchronized to a ∼7 h window, collected 10 hpi, 25.5 hpi, 39 hpi; PF3D7_1200700negative replicate 1 synchronized to a ∼9 h window, collected 12 hpi, 24 hpi, 41 hpi; PF3D7_1200700negative replicate 2 synchronized to a ∼10 h window, collected 12.5 hpi, 25 hpi, 41hpi).

#### ATAC-seq Library Preparation

Native parasite nuclei were isolated as in Bártfai et al. (2010). In short, after lysis of RBCs by 0.05% saponin treatment and separating nuclei from parasite debris using a cell lysis buffer (CLB: 10 mM Tris-HCL pH8.0, 10 mM NaCl, 3 mM MgCl_2_, 0.2% NP-40) with 0.25 M sucrose cushion. A 10 μL sized nuclei pellet was resuspended with a cut-off pipet tip in 337.5 μL CLB and for ATAC-seq replicate 1 69 μL of nuclei was used for t05 to t20 and 23 μL of nuclei was used for t25 to t40 (these volumes were based on previous tests using a dilution series of nuclei). For ATAC-seq replicate 2 we had to optimize the amount of nuclei again due to the use of a kit from a different lot and this led us to use 466 μL of nuclei for t05 and t10 and 155 μL for t15 and t20. Nuclei were brought to 10.5 μL in CLB and used in a 25 μL ATAC reaction based on Lara-Astiaso et al. (2014) with 2 μL Tn5 transposase and 12.5 μL TD buffer (Nextera DNA Library Prep Kit, #FC-121-1030, Illumina, USA). Reactions were incubated for 1 h in a 37°C heat block. Nuclei were kept in suspension by gently tapping the tube every 10 minutes. The reaction was stopped by addition of 5 μL clean up buffer (900 mM NaCl, 300 mM EDTA), 2 μL 5% SDS and 2 μL proteinase K (Sigma-Aldrich #P6556) and incubated for 30 min at 40°C. Tagmentated DNA fragments were isolated using 2.4 sample volume of Agencourt AMPure XP beads (Beckman Coulter, #A63882, USA). Half of the isolated DNA was used for library preparation (the other half was stored as back-up) starting with a size selection using 0.85x volumes of AMPure XP beads to enrich for fragments of 500 bp and smaller. Size-selected fragments were amplified using the KAPA HiFi HotStart ready-mix (KAPA Biosystems, #KK2602, US) and Nextera index primers (Nextera DNA Sample Preparation Index Kit, #FC-121-1012) under the following conditions: 98°C for 2 min; 16 cycles of 98°C for 20 s, and 62°C for 3 min; 62°C for 5 min. Libraries were purified using 1x volumes Agencourt AMPure XP beads. The fragment size distribution of the libraries was evaluated in a High-Sensitivity Bioanalyzer run (Agilent, #5067-4626, US) and the size selection was repeated when there was a large proportion of fragments longer than 500 bp (replicate 1 t05, t15, t30, t35, t40). To control for sequence bias, the same ATAC protocol was applied to genomic DNA from synchronous wild-type 3D7 *P. falciparum* ring stage parasites using 547.0 ng or 60.8 ng of input DNA. All ATAC-seq libraries were KAPA quantified (KAPA Library Quantification Kit, #KR0405).

#### RNA-seq Library Preparation

Parasite cultures were immediately placed on ice and washed once with ice-cold PBS. Pelleted cultures were resuspended in RLT buffer (QIAGEN, #74106) supplemented with 1% β-mercaptoethanol and snap-frozen in liquid nitrogen. Total RNA was extracted using the RNeasy Mini Kit (QIAGEN, #74106; including RNA clean-up and two on-column DNase treatments) and RNA concentration was measured using the Qubit RNA HS Assay Kit (Invitrogen, #Q32852). RNA was then polyA-selected using the Oligotex mRNA Mini Kit (QIAGEN, #70022) according to manufacturer’s instructions. Subsequently, 2000 ng of polyA-selected total RNA equivalent were fragmented by alkaline hydrolysis (40 mM Tris acetate pH 8.2, 100 mM potassium acetate,30 mM magnesium acetate) for 1 min 45 s at 85°C in a 150 μl volume and precipitated as previously described in Hoeijmakers et al. (2013). Next, polyA-selected RNA was cleaned from remaining genomic DNA (detected by qPCR) by two additional TURBO DNase treatments (Ambion, #AM2238). Strand-specific RNA-seq was performed as in Kensche et al. (2016). Accordingly, first strand cDNA synthesis was performed with AT-corrected Random N9 primers (76% AT) in the presence of 0.2 μg Actinomycin D (Thermo Fisher Scientific #11805017). During second strand synthesis dTTPs were substituted with dUTPs to preserve strand-specific information. Next, 10 ng of each double stranded cDNA library was end repaired, extended with 3′ A-overhangs, barcoded with NextFlex adapters (Bio Scientific, #514122) and treated with USER enzyme (NEB, #M5505L) to specifically degrade the dUTP-containing second strand. Libraries were amplified by PCR (98°C for 2 min; 4 cycles of 98°C for 20 s, 62°C for 3 min; 62°C for 5 min) using KAPA HiFi HotStart ready mix (KAPA Biosystems, #KM2602) and NEXTflex primer mix (Bio Scientific, #514122) and subsequently gel size-selected for 300 - 400 bp using 2% E-Gel Size Select agarose gels (Invitrogen, #G6610-02). After an additional eight cycles of amplification (see above), libraries were purified and adaptor dimers depleted using 1x volume of Agencourt AMPure XP beads (Beckman Coulter, #A63880).

#### Parasite Transfection

Parasite transfections were performed as in Fidock and Wellems (1997) using a BTX electroporation system. Synchronized ring stage 3D7 *P. falciparum att*B(+) parasites were pelleted by centrifugation and 100% hematocrit packed cells were mixed with 75 μg of the pINT and 75 μg of the desired *att*P*(+)_*min*kahrp* plasmid in cytomix (120 mM KCl, 0.15 mM CaCl_2_·2H_2_O, 5mM MgCl_2_·6H_2_O, 25 mM HEPES, 2 mM EGTA, 10mM K_2_HPO_4_, 10mM KH_2_PO_4_) in 450 μL total volume in a 2 mm electroporation cuvette (BTX, #45-0125). After transfection, parasites were resuspended in warm culture medium and cultured at 2.5% hematocrit in the presence of 2.6 nM WR99210 (Jacobus Pharmaceutical Company), 2.5 μg/ml Blasticidin S HCl (GIBCO, #R210-01) and 250 μg/ml Geneticin (GIBCO, #11811-031). After seven days, culturing was continued without Geneticin. When the parasite cultures became blood-smear, positive site-specific integration of the *att*P-containing plasmid into the parental line was confirmed by performing PCR using the primer combinations ‘p1_for’/‘p1_rev’ and ‘p2_for’/‘p2_rev’ (Table S7) on extracted genomic DNA (QIAGEN, #51106). Genomic DNA of the non-transfected parental 3D7 line was used as negative control (Figures S2A and S2B). Afterward parasites were cultured at a three week on/off schedule of 2.6 nM WR99210 and 2.5 μg/ml Blasticidin S HCl.

#### Plasmid DNA Cloning

To examine the regulatory potential of the identified accessible several parasite lines were generated: four parasite lines with an integrated plasmid containing an accessible region detected by ATAC-seq upstream of a minimal *kahrp* promoter and a *gfp-luc* reporter gene (Figures 4A and S2A), two parasite lines with a not-accessible, control region instead of the accessible region (a third line did not show successful integration) and one parasite with an integrated plasmid containing the minimal promoter followed by the reporter gene. To generate these parasites, we applied the Bxb1 integrase-mediated site-specific *att*P/*att*B integration system from Nkrumah et al. (2006) which results in directional integration of an *att*P-site containing plasmid into the *cg6* locus of a parental *att*B site-containing *P. falciparum* line. To generate the specific *att*P-plasmids, the pDC2 plasmid (Nkrumah et al., 2006) was modified on several points. (All primers used for cloning, integration checking and RT-qPCR are listed in Table S7). The orientation of the 5′*cam-snf7-gfp-3′hsp86* cassette was reversed using the primers ‘5′Pfcam-F’ and ‘3′hsp86-R’, PstI/*Apa*I digestion and ligation by the T4 ligase (Promega, #M1804) resulting in plasmid pOM1. The *snf7-gfp* element was replaced by the *gfp-luc* sequence from the MV163 plasmid (Vos et al., 2015) using the primers ‘GFPLuc-F’ and ‘GFPLuc-R’, *Avr*II/*Xho*I digestion and ligation by the T4 ligase resulting in plasmid pOM2. Finally, the *5′cam* was replaced by the *kahrp* minimal promoter (Brancucci et al., 2012) using the primers ‘kahrp-F’ and ‘kahrp-R’, digestion by *Avr*II/*Age*I and T4 ligation resulting in plasmid *att*P*(+)_*min*kahrp*. Accessible or control regions located upstream of the genes PF3D7_0719000, PF3D7_1200700 and PF3D7_1222700 or the accessible region upstream of PF3D7_1372200 were amplified and inserted upstream of the *kahrp* minimal promoter using their respective primers listed in Table S7 and BglII/*Not*I digestion and ligation by the T4 ligase. These accessible regions were selected because they showed clear, distinct, stage-specific accessibility patterns; the downstream gene showed a matching gene expression pattern; we favored accessible regions located in tandem intergenic regions (i.e., containing a single promoter) for clarity of the assignment between genes and accessible regions; and we excluded ATAC regions and parts of the peak that overlapped with a TSS.

#### RNA Extraction, cDNA Synthesis and qPCR

Total RNA was extracted as described in “RNA-seq library preparation” and was checked for genomic DNA contamination by qPCR. If needed, the sample was additionally treated once or twice with TURBO DNase (Ambion, #AM2238). For each sample 500 to 1000 ng of total RNA was mixed with random hexamer primers (0.5 μg, Roche #11034731001), OligodT_12-18_ (0.5μg, Invitrogen #18418012) and dNTPs (0.5mM in the final volume of 20μl, Invitrogen 10297-018) and incubated for 5min at 70°C. First strand synthesis was performed for 1h at 42°C in First Strand Buffer (Invitrogen) supplemented with DTT (10 mM), Superscript III (200 units, Invitrogen, #18080044) and RNasin Plus RNase inhibitor (40 units, Promega, #N261B), after which superscript III was inactivated by incubation at 70°C for 15min. For all samples, a negative control reaction was performed in which Superscript III was replaced by water (RT minus control) under identical conditions. For each parasite line the same amount of RNA was used as template from the different time points.

To measure the relative *gfp-luciferase* (*gfp-luc*) transcript abundance, a qPCR was performed using SYBRgreen supermix (BioRad) and primers which were mixed according to the manufacturer’s instructions. The qPCR was preformed using the CFX96 Real Time Systems C1000 Touch Thermal Cycler (Bio-Rad) with the following program: 95°C for 3min, (94°C for 10 s, 52°C for 30 s, 68°C for 30 s) 39 cycles, 95°C for 1min, 65°C for 1min and a gradient from 65°C to 94.5°C with a 0.5°C increase every 10s. Primers specific for *gfp-luc* served to assess the relative abundance of the reporter transcript (‘GFP-1’, ‘GFPLuc’, ‘Luc-1’. ‘Luc-2’) and primers for *blasticidin* and *actin* (‘BSD-1’, ‘BSD-2’, ‘actin’) controlled for successful cDNA synthesis (data not shown). All -RT controls reported ‘not detectable’ (NA) or in Cq values in the range of the H_2_O control, which was included for all primer pairs (data not shown). The relative *gfp-luc* transcript abundance was measured against a standard dilution series prepared from *P. falciparum* 3D7 *att*B(+) genomic DNA and pOM2 plasmid DNA mixed in a close to 1 molar ratio (10-fold dilution series of genomic DNA ranging from 5 pg – 5000 pg, 10-fold dilution series of plasmid DNA ranging from 0.005pg - 5pg). As different standard series were used for the positive replicate 1 and replicate 2, the data of replicate 2 was scaled to the average of replicate 1 for Figure 4. The raw data for each replicate are depicted in Figure S2B.

#### Nuclear Protein Extract Generation and DNA Pull-down

For collections, asynchronous asexual *P. falciparum* 3D7 cultures were put on ice immediately and filtered over Plasmodipur filters (EuroProxima, Netherlands) to remove human white blood cells. Infected RBCs were washed once in PBS and resuspended in PBS with Protease Inhibitor Cocktail (PI at 1:100, Roche, #04693132001) and 0.05% saponin to a maximum of 6.25% hematocrit for a maximum of 15 minutes. Nuclei were isolated over a double sucrose gradient in CLB with PI (PI at 1:50, bottom layer of 0.25 M sucrose, top layer 0.1 M sucrose) and resuspended in CLB with 20% glycerol, pelleted by centrifugation, snap-frozen and stored at −80°C until the generation of the nuclear protein extract. Nuclear protein extract was generated as in Kensche et al. (2016) with two rounds of extraction in High Salt Extraction Buffer (50 mM HEPES pH7.5, 20% glycerol, 420 mM NaCl, 1.5 mM MgCl_2_, 1 mM DTT, 0.4% NP-40, PI). Protein concentration was measured using a Qubit fluorometer (Qubit Protein Assay Kit, Thermo Fisher Scientific, #Q33212). Nuclear protein extract was snap-frozen in aliquots and stored at −80°C. Right before the pull-down, nuclear protein extracts were diluted to 0.909 mg/ml protein concentration in 50 mM HEPES pH7.5, 10% glycerol, 150 mM NaCl, 1.5 mM MgCl_2_, 1 mM DTT, 0.125% NP-40, PI at 1:25, 9 ng/μl yeast tRNA (Sigma-Aldrich, #R5636), 9 ng/μl poly(dI:dC) (Sigma-Aldrich, #P4929) and 9 ng/μl poly(dA:dT) (Sigma-Aldrich, #P0883). Diluted extracts were spun once at 17000 x g for 25 minutes at 4°C to remove precipitates.

DNA pull downs were performed as in Hubner et al. (2015) and Kensche et al. (2016). Probes for DNA pull downs (ordered from Integrated DNA Technologies, US; Table S7) were dissolved in TE (10 mM Tris, 0.1 mM EDTA, pH 8.0) to 200 μM. 1000 pmoles of biotinylated forward probe was annealed to 1500 pmoles of reverse probe in annealing buffer (10 mM HEPES pH 8.0, 0.05 M NaCl, 1 mM EDTA, in DNase free water). For each pull-down, 50 pmoles of dsDNA probe was coupled to 10 μL of washed Sepharose beads slurry (GE Healthcare, #17511301) in DNA Binding Buffer (DBB: 10 mM HEPES pH 8.0, 1 M NaCl, 10 mM EDTA, 0.05% NP-40 in DNase free water) in a total volume of 350 μL while rotating at RT for at least 1 h. Excess probes were removed by two washes with 500 μL DBB and two with 500 μL Protein Binding Buffer^∗^ (PBB^∗^: 50 mM HEPES pH 8.0, 150 mM NaCl, 0.1% NP-40, 1 mM DTT, PI at 1:25). After the last wash, PBB^∗^ was removed almost completely for each reaction and 550 μL of diluted nuclear protein extract with 500 μg protein content was added and incubated for 1.5 h while rotating at 4°C. Reactions were spun at 400 x g and supernatants were discarded. Beads (with probes and bound proteins) were then washed by once with 1 mL PBB^∗^, twice with 1 mL PBB (PBB^∗^ without PI) and twice with 1 mL Wash Buffer (WB: 50 mM HEPES pH 8.0, 150 mM NaCl). After the last wash with PBB and the washes with WB, supernatants were removed as much as possible. Disulfide bonds were reduced by incubating the beads with 5 mM TCEP (Sigma-Aldrich, #C4706-2G dissolved to 100 mM in mass-spec grade Milli-Q and stored at −20°C) in 100 mM TEAB (Sigma-Aldrich, #T7408-100 ml) for 1 h in a 37°C shaking heat block. Beads were briefly spun down and incubated with 10mM (final concentration) of MMTS (Thermo Scientific, #23011, dissolved to 200 mM in isopropanol and stored at −20°C) to alkylate disulfide bonds in a 37°C shaking heat block for 10 min. Beads were briefly spun down and 0.4 μg Trypsin/LysC (dissolved to 0.4 μg/μl in Resuspension buffer (50 mM acetic acid (pH < 3), Promega, #V5072) was added and incubated for 1 h in a 37°C shaking heat block. Beads were spun for 1 min at 200 x g at RT and supernatants were collected in a new Eppendorf tube. 50 μL of 100 mM TEAB was added to the beads and these were incubated for another 5 min in a 37°C shaking heat block and supernatants were added to the previously collected supernatants. Trypsin digestion in the supernatants was continued by overnight incubation in a 37°C waterbath. Each probe was tested twice per experiment and peptides were labeled by dimethyl labeling (Boersema et al., 2009). NaBH_3_CN (Merck, #818053) and CH_2_O were used for ‘light’ labels and NaBD_3_CN (Sigma-Aldrich, #190020-1G) and CD_2_O for the ‘heavy’ labels. Labeling reactions were incubated for 1 h at RT while shaking and labeling was stopped by addition of 16 μL of 1% ammonia. Reactions of wild-type and mutated probes with different labels were then pooled and acidified by addition 10 μL of 100% trifluoroacetic acid (TFA, Biosolve BV, the Netherlands, #20234131). Samples were then cleaned and concentrated on C18 stage tips (Rappsilber et al., 2007) and stored at 4°C until measurement.

### Quantification and Statistical Analysis

#### ATAC-seq Data Analysis

The ATAC-seq libraries were sequenced for 75 bp, paired-end on a NextSeq500 system (Illumina) using NextSeq500/550 HighOutput kit V2 (75 cycles) reagents (Illumina). Raw fastq reads were first evaluated using FastQC before continuing (Andrew, 2010) and reads obtained from the two gDNA control libraries were combined after sequencing. Paired-end libraries were mapped with BWA-mem (version 0.7.10; Li, 2013) against the *P. falciparum* 3D7 reference genome (PlasmoDB release 26; Aurrecoechea et al., 2009, Logan-Klumpler et al., 2012) and filtered for mapping quality > = 30 (samtools version 1.3.1; Li et al., 2009). Duplicate reads were removed using Picard tools (version 1.139; Broad Institute, https://broadinstitute.github.io/picard/) and reads mapping to the apicoplast and mitochondrial DNA were removed as well as supplementary alignments (FLAG 2048). Finally, an *in silico* size selection was performed to select for read pairs with insert sizes between 50 and 150 bp (or different when indicated) and these libraries were used for further analysis (between 5.9 and 9.7 million reads for replicate 1; between 3.6 and 6.4 million per library for replicate 2; 36.9 million for the merged gDNA control library). For visualization, these libraries were converted to bedgraph files using bedtools genomecov (version 2.20.1; Quinlan and Hall, 2010) with the option ‘-pc’ for paired end data and scaled per million reads (RPM). Alternatively, for genomic DNA-corrected tracks, the coverage in each of the t05 to t40 libraries (with an offset of +0.1) was divided by the coverage in the gDNA library (with an offset of +0.1). Bedgraph files were visualized in the UCSC genome browser (Kent et al., 2002).

Downstream analyses were performed using the data from ATAC-seq replicate 1. Peak calling we used the MACS2 subcommands ‘macs2 pileup’, ‘macs2 bdgcmp’ and finally ‘macs2 bdgpeakcall’ (MACS2 release 2.7; Liu, 2016). Because some MACS2 subcommands cannot handle paired end data, we first binned the libraries based on the insert size in steps of 5 bp. Next, the start site of reads aligning to the positive strand were shifted with +4 bp and those aligning to the minus strand with −5 bp to represent the center of the Tn5 transposon binding event as in Buenrostro et al. (2013). Then a pileup track for each (binned) ATAC library was calculated by MACS2 pileup with–extsize set to half the mean insert size. The pileup tracks of the binned libraries were then summed, scaled per million of reads and a pseudocount of 0.1 was added to every position. Regions of local enrichment were identified with macs2 bdgcmp using the gDNA pileup track as background and scored in qvalues (-m qpois). Finally, macs2 bgpeakcall was used to identify regions with qvalue below 0.001 (-c 3.0). To prevent calling many small ‘peaks’ we allowed regions to be merged when they were within the maximum insert size of 150 bp (-g 150) and we set the minimum length of a peak to 100 bp (-l 100).

Peaks for all time points were merged and the highest scoring summit was selected as summit for the merged peak (4035 merged peaks in total). Peaks with a summit located in a coding region (209 merged peaks) or more than 3kb away from the first/last gene on each chromosome (71 merged peaks) were removed from further analyses. To assign the remaining 3755 intergenic peaks to genes we only selected peaks in intergenic regions flanking the 5′ of a gene. For this purpose, intergenic regions (IGs) were categorized based on the flanking coding sequences: ‘tandem IGs’ are flanked by two genes both in 5′→ 3′ or in 3′←5′ orientation; ‘divergent IGs’ have a downstream gene with 3′←5′ orientation and an upstream gene in 5′→3′ orientation; ‘convergent IGs’ have a downstream gene in 5′←3′ orientation and the upstream gene in 3′→5′ orientation (Figure 1C). Peaks with their summit located in ‘tandem IGs’ and ‘divergent IGs’ (3647 peaks) were assigned to the closest downstream gene using bedtools closest (version 2.20.1; Quinlan and Hall, 2010). To calculate accessibility per stage, tags were counted for each of the merged peaks in tandem or divergent IGs. Tag counts were offset by +1 and normalized to the number of reads per kb per million mapped reads (RPKM). For each peak the maximum RPKM value was determined across the stages and peaks with the lowest 10% of maximum values were removed. To correct for signal intensity differences among the time points we normalized the data on quantiles (using the normalized.quantiles command from the R package preprocessCore version 1.36.0; Bolstad, 2017). Then, for each peak we calculated the proportion of signal per time point compared to the summed signal over all time points. This proportion-of-sum value was used to calculate the accessibility pattern in each peak region over the time course (t05 – t40).

#### Directional RNA-sequencing

Strand-specific RNA-seq libraries were sequenced on the Illumina NextSeq 500 system to obtain 75 bp single-end reads (NextSeq500/550 HighOutput kit V2 (75 cycles) reagents (Illumina)). Reads were evaluated using FastQC (Andrew, 2010) and mapped against the annotated *P. falciparum* 3D7 transcriptome from PlasmoDB release 26 (Aurrecoechea et al., 2009, Logan-Klumpler et al., 2012) using BWA samse (version 0.7.12-r1039; Li and Durbin, 2010). Single-end reads were filtered to mapping quality ≥ 15 (samtools version 1.2; Li et al., 2009) and only uniquely mapped reads (between 9.2 and 11.6 million per library) were used for further analysis. To visualize RNA-seq data in the UCSC Genome browser, 75bp reads were additionally mapped against the annotated *P. falciparum* 3D7 genome from PlasmoDB version 26 (Aurrecoechea et al., 2009, Logan-Klumpler et al., 2012), filtered for uniquely mapped reads and mapping quality ≥ 15. Reads were separated according to the strand they mapped to (sense strand FLAG16, antisense strand FLAG0) and normalized to the number of mapped reads per million (RPM). Bedgraph files were generated (version 2.20.1; Quinlan and Hall, 2010) and visualized in the UCSC genome browser (Kent et al., 2002).

To assess RNA abundance per gene, reads mapped against the transcriptome were separated based on alignment to the sense (FLAG 16) or antisense strand (FLAG 0) respectively. Only reads aligning to the sense strand of each transcript were used for further analysis. Tags were counted for all transcripts (excluding mitchochondrial RNA and apicoplast RNA) and offset by +1. Transcript counts were normalized to the number of reads per kb per million mapped reads (RPKM) and the maximum RPKM value was determined per transcript. Low abundant transcripts with their maximum RPKM value across the stages were discarded (lowest 10 percentile). Relative transcript abundance to assess stage-specific expression patterns over the time course (t05 – t40) was calculated by dividing the RPKM value of each time point through the sum of RPKM values of all time points (proportion of sum).

#### Comparison of ATAC-seq and RNA-seq Data

To compare accessibility and transcript abundance patterns, accessibility (proportion-of-sum) was clustered using the web-based Morpheus tool from the Broad Institute (https://software.broadinstitute.org/morpheus/) into eight clusters by k-means clustering with the 1-pearson correlation coefficient as distance metric and 20.000 iterations. Relative transcript abundances (proportion-of-sum) of the downstream gene were then plotted in the same order. Accessibility and mRNA abundance profiles were visualized on a heatmap using color scale covering the 20^th^ to 80^th^ percentile of values. The Pearson correlation coefficient was calculated for each peak-to-transcript pair. Randomized correlations were calculated for 1000 shuffled peak-to-transcript matches.

For co-clustering of accessibility and transcript abundance patterns, peak-to-transcript matches were first filtered for a Pearson correlation coefficient above 0.6. The resulting matrix of accessibility and transcript abundance data (n = 2118 matches) was uploaded in Morpheus and again clustered into 8 k-means with the same settings as before.

#### Comparison with AP2-I ChIP-sequencing Data

For visualization purposes, the AP2-I ChIP-sequencing data from Santos and co-workers was mapped against the *P. falciparum* 3D7 genome with settings as in Santos et al. (2017). In short, reads were trimmed with Trimmomatic (version 0.36; Bolger et al., 2014), mapped with BWA-mem (version 0.7.10; Li, 2013) against the *P. falciparum* 3D7 genome (PlasmoDB release 26; Aurrecoechea et al., 2009, Logan-Klumpler et al., 2012) and filtered for not being the primary alignment (FLAG 256), being a duplicate (FLAG 1024), being a supplementary alignment (FLAG 2048) and for mapping quality of 30 and higher. The MACS2 callpeak command was used to generate bedgraph files of the ChIP and input libraries (settings–m 5 50–extsize 250–call-summits -B -q 0.05 -g 2.2e7). These bedgraph files were used to make log2 ChIP-over-input tracks that were uploaded in the UCSC genome browser. Bedtools intersect (version 2.20.1; Quinlan and Hall, 2010) was used to define the overlap between the ATAC-seq peaks with the trimmed AP2-I peaks (in 3D7 coordinates) reported in Santos et al. (2017). Fluff was used to generate the heatmap of accessibility over the AP2-I peaks that overlap with ATAC-seq peaks (Georgiou and van Heeringen, 2016). We used the build-in Gene Ontology tool of PlasmoDB with default settings to identify enriched GO terms (Aurrecoechea et al., 2009).

#### Motif Identification and Enrichment Analyses

For *de novo* motif identification we used gimme motifs from the GimmeMotifs package (v0.11.0; van Heeringen and Veenstra, 2011). Numerous *de novo* motif searches were performed on individual time points and using different number of clusters ranging from 4 to 16 in regions of 200 or 300 bp around the summit. The background consisted of either shuffled peak regions or the other clusters. Searches were performed for large (6 - 15 bp) or xl (6 - 20 bp) motifs. Motifs identified in these various searches were clustered using gimme cluster (-t 0.9) yielding a non-redundant list of *de novo* motifs. To identify motifs differentially enriched in one of the 8k means co-clusters of the ATAC-seq and RNA-seq data compared to other co-clusters, we run an ensemble of different regression and classification methods, as implemented in GimmeMotifs (van Heeringen and Veenstra, 2011). As input motif library we used the clustered *de novo* motifs, predicted *Plasmodium* motifs (Campbell et al., 2010, De Silva et al., 2008), and motifs from plants, vertebrates and invertebrates reported in CISBP (Weirauch et al., 2014), motifs from each subgroup were first clustered with gimme cluster at -t 0.9). Gimme maelstrom was ran three times and we selected motifs that had a P value = < 0.01 in at least two runs. From this list we manually removed eight low information content motifs. The remaining 70 motifs were grouped based on their similarities by gimme cluster (-t 0.9), resulting in 41 ‘motif groups’.

#### Mass Spectrometry and MS Data Analysis

Loaded C18 stage tips were rehydrated with 25 μL buffer A (0.1% formic acid) and peptides were eluted in PCR tubes using 30 μL buffer B (80% acetonitrile, 0.1% formic acid). Acetonitrile was evaporated by a 15 min vacuum spin at room temperature and samples were reconstituted to 12 μL with buffer A of which 5 μL measured on a QExactive or Orbitrap Fusion mass spectrometer (Thermo Fisher Scientific). In both cases, the sample was separated over a 30cm C18-reverse phase column (1.8 μm Reprosil-Pur C18-AQ, dr. Maisch 9852) and eluted using an Easy-nLC 1000 (Thermo Fisher Scientific). For the QExactive, elution was preformed over a 94 min gradient (5.6% acetonitrile/0.1% formic acid - 25.6% acetonitrile/0.1% formic acid) and directly injected into the mass spectrometer. Data on the QExactive was acquired in TOP10 data-dependent acquisition mode with dynamic exclusion enabled for 20 s. Resolution for MS was set at 70.000 at m/z = 400 and for MS/MS at 17.5000. For the Fusion, elution was performed over a 114 min gradient (7.2% Acetonitrile/0.1% formic acid- 25.6% acetonitrile/0.1% formic acid) and directly injected into the mass spectrometer. Data on the Fusion was acquired in data-dependent top speed mode in a 3 s cycle with dynamic exclusion set at 60 s. Resolution was set at 120.000.

Raw MS spectra were analyzed as in Kensche et al. (2016) using MaxQuant (version 1.5.3.30; Cox and Mann, 2008). In short, standard settings were applied with the following modifications. Multiplicity was set at 2, adding a mass of 28.03 Da (light-labeled) or 36.08 Da (heavy-labeled) to the peptides N terminus and lysine residues. Trypsin/P was set as the specific digestion mode with maximum 2 missed cleavages. Analyses were run with re-quantify set to ‘match from and to’. MMTS (added mass of 45.99 Da) was specified as fixed modification of cysteines. The match-between-runs option was activated (with 0.7 min match time window and 20 min alignment time window) and calculation of iBAQ values was enabled. Peptide masses were searched against the *Plasmodium falciparum* 3D7 annotated proteome (PlasmoDB release 9.3; Aurrecoechea et al., 2009, Logan-Klumpler et al., 2012) with the entire human proteome included in the contaminants list using the integrated Andromeda search engine. Mass tolerance was set at 4.5 ppm for precursor ions and 20 ppm for fragment ions, and peptides and proteins were accepted with an 0.01 FDR cut-off. Protein quantification was set to minimally require a single peptide-ratio, but a more stringent downstream filtering on minimally 2 peptides (of which at least 1 unique) was applied for generation of scatterplots and determination of significance.

Downstream analyses were performed using the Perseus software package (version 1.4.0.20; Tyanova et al., 2016). Normalized H/L-ratios were log2-transformed and intensity values were log10-transformed. Significant outliers were determined using the intensity-based Significance B option (two-sided Benjamini-Hochberg test) with a FDR cut-off set to 0.05. The protein list was filtered for reverse hits, proteins that are only identified by site and potential contaminants. In addition, proteins required a minimum of 2 peptides of which at least 1 unique in order to be considered as a hit in both the forward and reverse experiment. Data was plotted in R and significant outliers were labeled. Candidate TFs were retrieved from Table 4 in Bischoff and Vaquero (2010) and highlighted as well.

### Data and Software Availability

The accession number for the ATAC-seq and RNA-seq data reported in this paper is GEO: GSE104075.

## Supplemental Information

Document S1. Figures S1–S5

Table S1. Genome Coordinates of ATAC Peaks, Related to Figures 1, S1, and 5

Table S2. Fluff Heatmap Clustering AP2-I Peaks Using ATAC-Seq Data, Related to Figure 2

Table S3. Result of the Motif Enrichment Analysis and Associated Position Weight Matrices, Related to Figures 5 and S3

Table S4. Proteins Identified in DNA Pull-Downs, Related to Figures 6 and S4

Table S5. Parasite and Bacterial Strains, Related to STAR Methods

Table S6. Parasite Staging for Time-Course Experiments (Replicate 1 and 2), Related to STAR Methods

Table S7. Primers and Oligos Used for Cloning, Integration PCR, RT-qPCR and DNA Pull-Down Experiments, Related to STAR Methods

Document S2. Article plus Supplemental Information
