# Supplementary material for: Chromatin Accessibility-Based Characterization of the Gene Regulatory Network Underlying Plasmodium falciparum Blood-Stage Development
Source: Cell Host Microbe. 2018 Apr 11;23(4):557–569.e9. doi: 10.1016/j.chom.2018.03.007 (PMC5899830; doi:10.1016/j.chom.2018.03.007)
Supplement: Document S1. Figures S1–S5 [file mmc1.pdf]

Cell Host & Microbe, Volume *23*

Supplemental Information

Chromatin Accessibility-Based Characterization of the Gene Regulatory Network Underlying *Plasmodium falciparum* Blood-Stage Development

Christa Geeke Toenhake, Sabine Anne-Kristin Fraschka, Mahalingam Shanmugiah Vijayabaskar, David Robert Westhead, Simon Jan van Heeringen, and Richárd Bártfai

Chromatin accessibility-based characterization of the gene regulatory network underlying *Plasmodium falciparum* blood stage development

Christa Geeke Toenhake, Sabine Anne-Kristin Fraschka, Mahalingam Shanmugiah Vijaya-Baskar, David Robert Westhead, Simon Jan van Heeringen and Richárd Bártfai

Supplemental Information

**Figure S1. ATAC-seq data processing and reproducibility. Related to Figure 1.**
**Figure S2. Reporter constructs containing *gpf-luc* gene under the control of accessible or control regions. Related to Figure 4.**
**Figure S3. Identification of putative DNA motifs. Related to Figure 5.**
**Figure S4. DNA pull downs for identification of potential protein interactors. Related to Figure 6.**
**Figure S5. Representative images of Giemsa smears from time-course experiment (replicate 1). Related to STAR Methods.**
**Table S1. Genome cooridnates of ATAC peaks. Related to Figure 1, Figure S1 and Figure 5.**
**Table S2. Fluff heatmap clustering AP2-I peaks using ATAC-seq data. Related to Figure 2.**
**Table S3. Result of the motif enrichment analysis and associated position weight matrices. Related to Figure 5 and Figure S3.**
**Table S4. Proteins identified in DNA pull downs. Related to Figure 6 and Figure S4.**
**Table S5. Parasite and bacterial strains. Related to STAR Methods.**
**Table S6. Parasite staging for time-course experiments (replicate 1 and 2). Related to STAR Methods.**
**Table S7. Primers and oligos used for cloning, integration PCR, RT-qPCR and DNA pull down experiments. Related to STAR Methods.**

Figure S1 Related to Figure 1

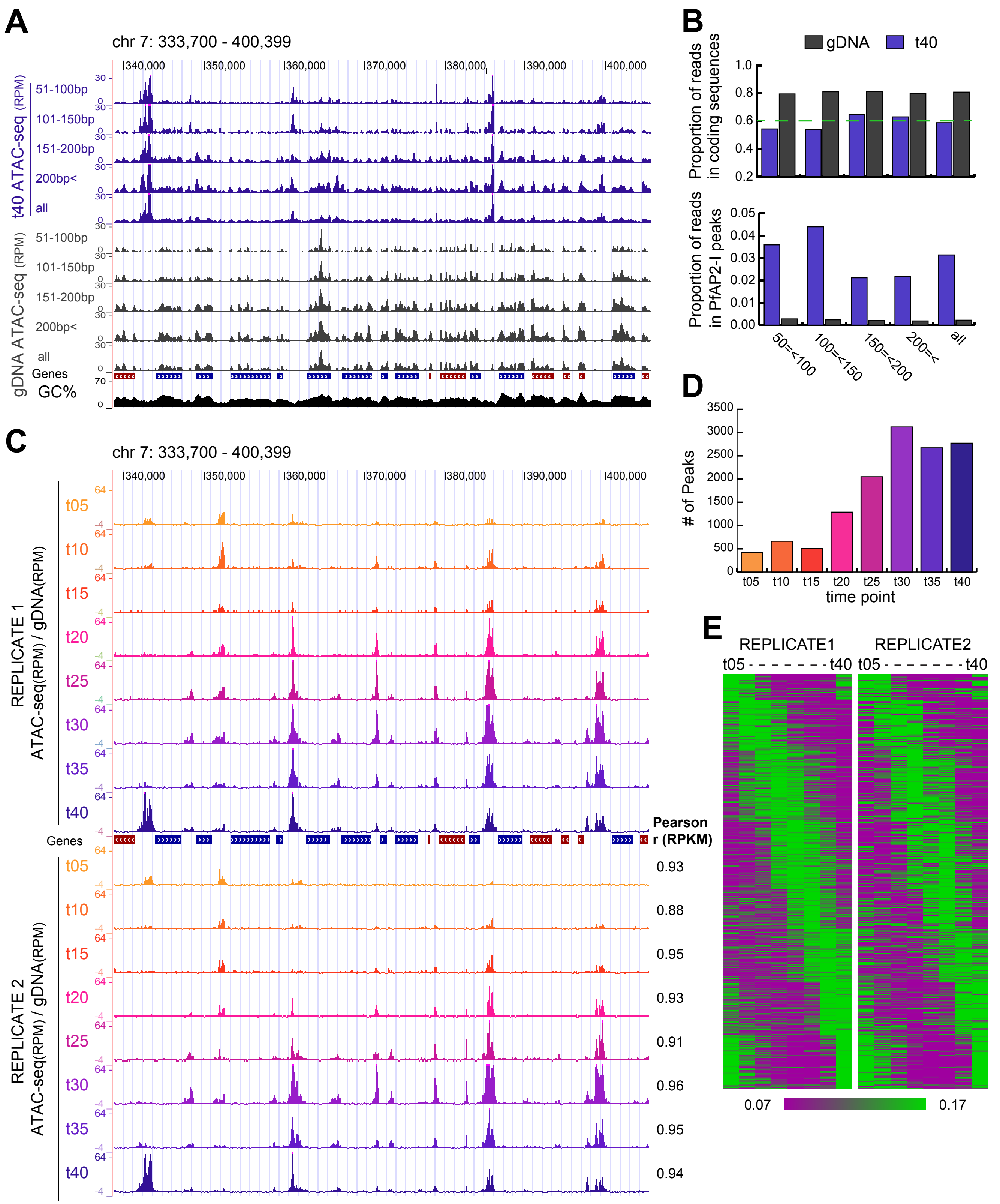

Figure S1. ATAC-seq data processing and reproducibility. Related to Figure 1.

A) UCSC genome browser screenshot of the region on chromosome 7 (333,700 - 400,399bp) showing libraries of t40 and gDNA without ('all') and after *in silico* selection for the indicated insert size ranges. Coding sequences are indicted as blue (positive strand) or red (minus strand) bars. GC%, the mean percentage of GC nucleotides, smoothened over 5 bp.

B) Barplots depict the proportion of reads located in coding sequences (top plot) or in AP2-I peaks (bottom plot) for t40 and gDNA ATAC-seq libraries without ('all') and after *in silico* selection for the indicated insert size ranges.

C) UCSC genome browser screenshot as in Figure 1A showing the gDNA-corrected ATAC-seq profiles for the two replicates over eight timepoints of blood stage development

D) Barplot of the number of peaks as called by MACS2 at each point.

E) Heatmaps of relative chromatin accessibility in the two ATAC-seq datasets for the regions and clustering depicted in Figure 3B. Relative accessibility was calculated as a proportion-of-sum of qnRPKM values over the time points and clustered by k-means using the 1-Pearson correlation distance metric. Color scales range from the 20th to the 80th percentile for both datasets.

Figure S2 Related to Figure 4

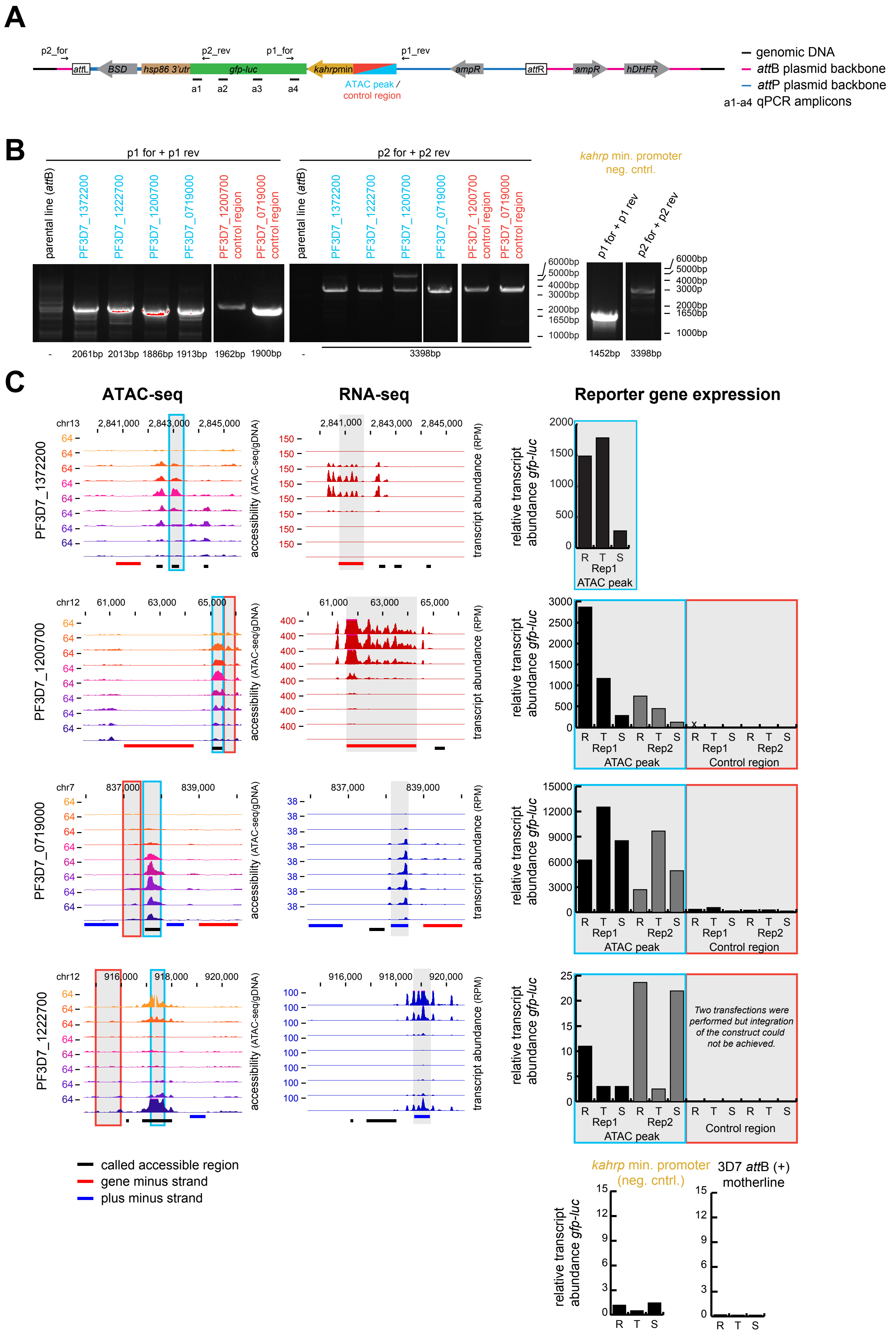

Figure S2. Reporter constructs containing *gpf-luc* gene under the control of accessible or control regions. Related to Figure 4.

A) Schematic representation of recombinant *cg6* locus (black) in the *att*B-containing parental *P. falciparum* 3D7 parasite line (Nkrumah et al., 2006) after integration with an *att*P-containing plasmid carrying the *gfp-luc* gene (green) under the control of the *kahrp* minimal promoter (yellow) with or without accessible or control region (light blue/orange). The *att*B plasmid backbone is indicated as a pink line, the *att*P integration plasmid backbone is blue. The position of primer pairs (p1 forward and reverse covering the accessible region and *kahrp* promoter or p2 forward and reverse covering the *gfp-luc* gene and plasmid backbone) used to confirm the generated parasite lines (see B) are indicated above the drawing. Location of amplicons (a1-4) measured by RT-qPCR (see C) are indicated below. The drawing is not to scale.

B) Confirmation of insertion of the DNA constructs to the *cg6* locus by PCR. Genomic DNA from *att*B-site containing parental parasites served as negative control.

C) UCSC genome browser screenshots showing gDNA-corrected ATAC-seq tracks and directional RNA-seq tracks (left and middle panel) for all eight time points. In the ATAC-seq screenshots, the cloned accessible regions upstream of PF3D7_0719000, PF3D7_1200700, PF3D7_1222700 and PF3D7_1372200 are shaded in grey with a blue rectangle. Selected control regions are highlighted with a dark orange rectangle. In the RNA-seq screenshots the relevant gene is shaded in grey. Black bars represent the called ATAC peaks. Red and blue bars are genes encoded on minus and plus strand. Right panel: Relative *gfp-luc* transcript abundance measured by qRT-PCR for ring, trophozoite and schizont stages from parasites containing the accessible (or control) region assigned to the PF3D7_0719000, PF3D7_1200700, PF3D7_1222700 or PF3D7_1372200 gene upstream of the minimal *kahrp* promoter. Note that the replicate 2 schizont sample for PF3D7_1222700 has been collected at a later time point, explaining the

higher reporter gene expression observed. The relative *gfp-luc* transcript abundance was assessed by taking the average of the four different primer pairs (see A, a1-4). ‘x’depicts missing value.

Figure S3 Related to Figure 5

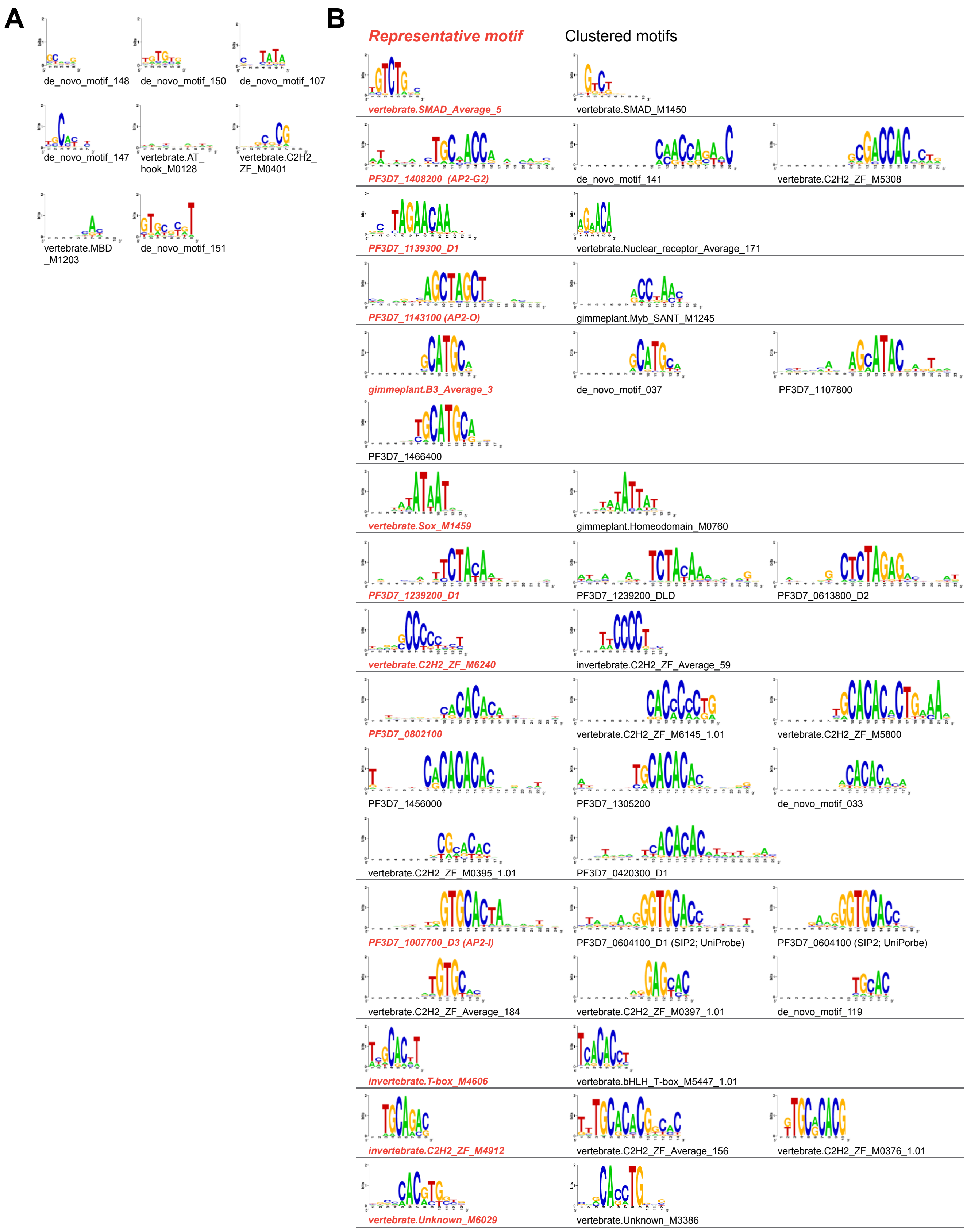

Figure S3. Identification of putative DNA motifs. Related to Figure 5.

A) List of eight repetitive and/or low-information content motifs excluded from further analyses.

B) Motif groups generated by clustering similar motifs found to be differential enriched (P =< 0.01 from rank aggregation test) for one or more ATAC/RNA-clusters. Representative motifs used in Figure 5 are indicated with red fonts.

Figure S4 Related to Figure 6

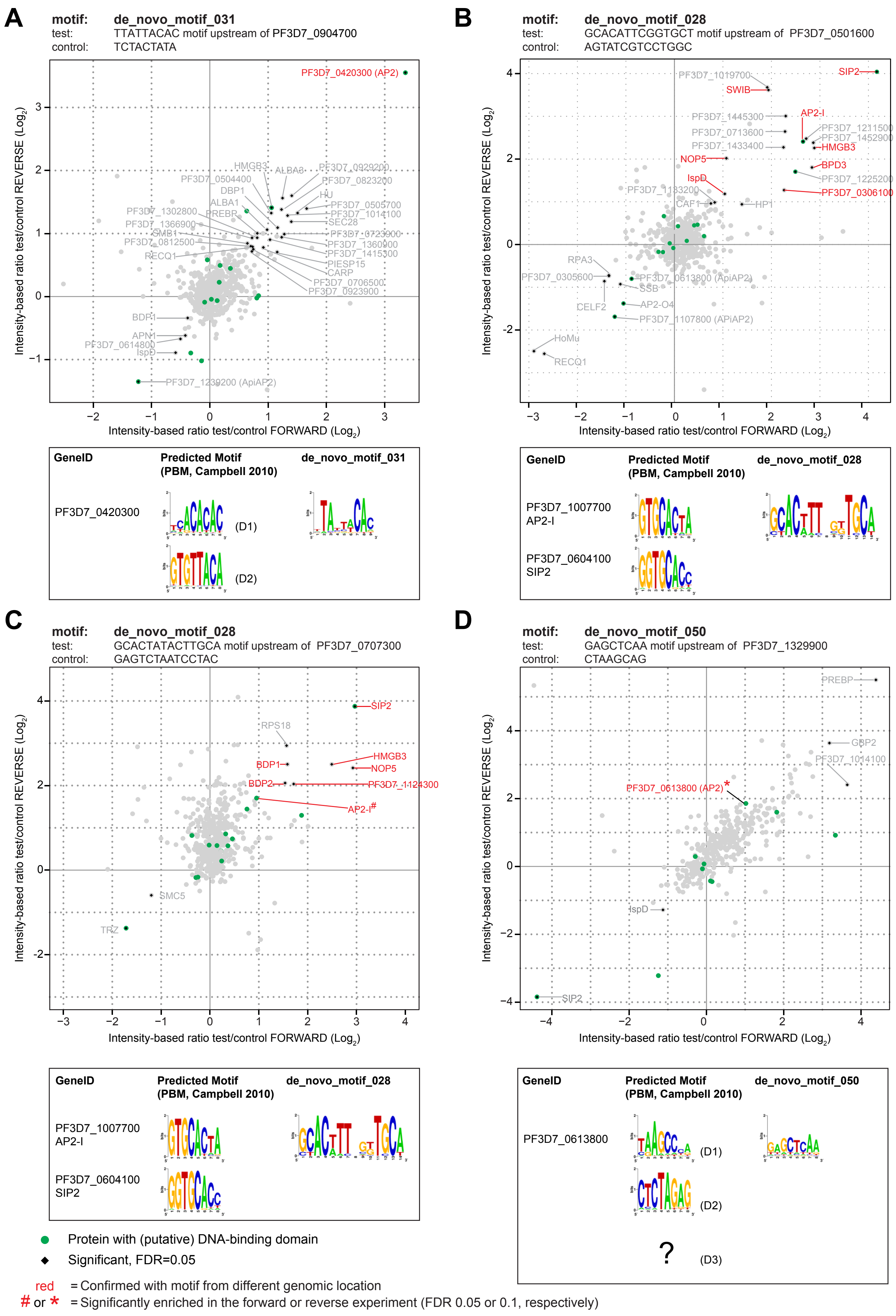

Figure S4. DNA pull downs for identification of potential protein interactors. Related to Figure 6.

Duplicate DNA pull downs with label swap performed using 60bp DNA probes with the (A) de_novo_motif_031 (TTATTACAC); (B-C) de_novo_motif_028 (GCACWWTNNKTGCW) or (D) de_novo_motif_050 (GAGCTCAA). The same probes with a scrambled motif were used as controls. The statistically significant outliers (black diamond, intensity-based FDR < 5%) are the proteins that interact with the motifs. Red font indicates that the interaction was confirmed using a probe from a different genomic region but containing the same motif. # and * indicate that the protein was significantly enriched in either the forward or reverse experiment at an FDR of 0.05 or 0.1, respectively. Green dots mark candidate DNA-binding factors derived from Table 4 of (Bischoff and Vaquero 2010).

Figure S5 Related to STAR Methods

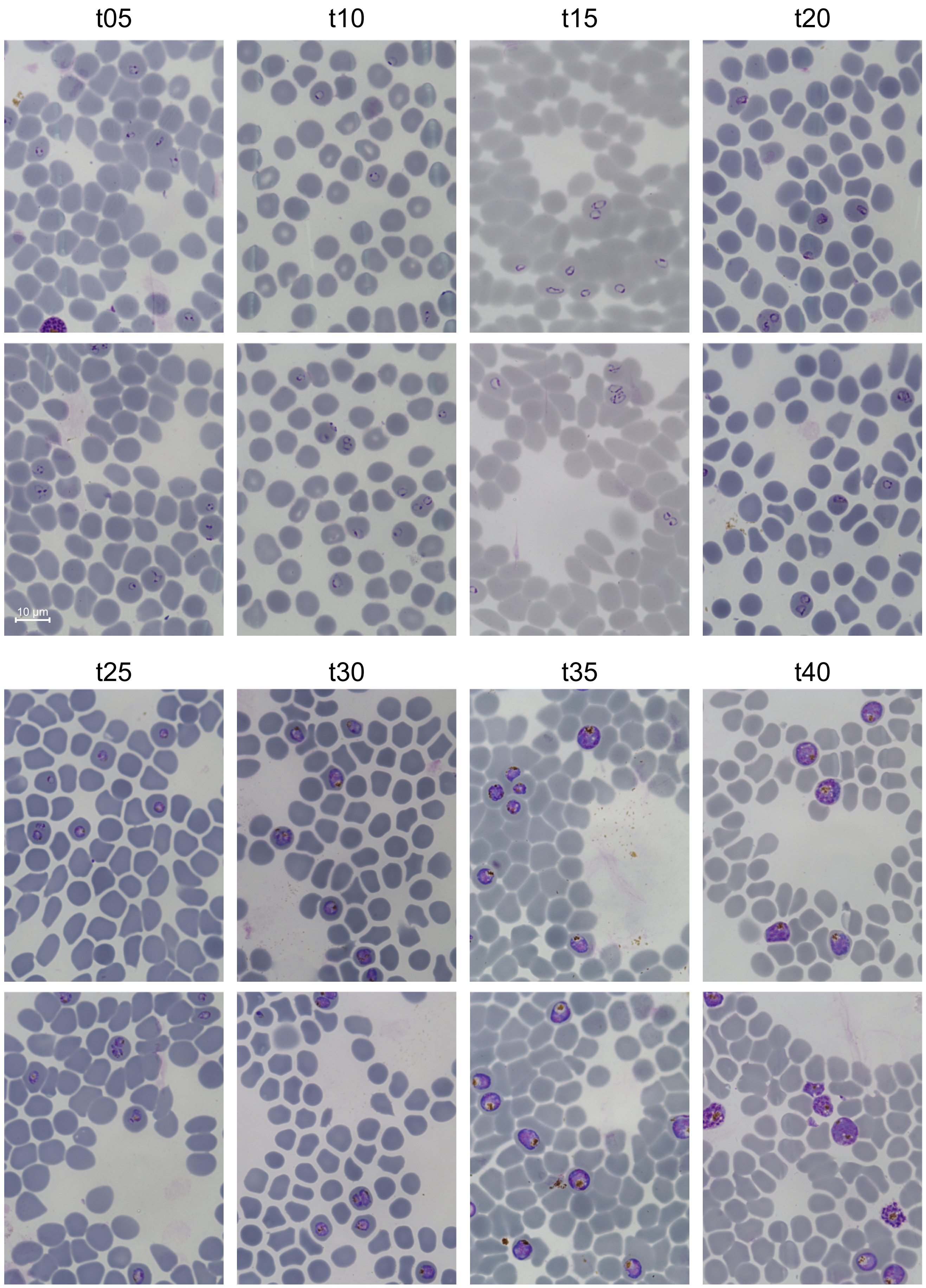

Figure S5. Representative images of Giemsa smears from time-course experiment (replicate 1). Related to STAR Methods.

For each time point in the time-course experiment Giemsa stained blood smears were prepared. The two images show the representative parasite morphologies from each time point. The scale bar in the bottom image of t05 represents 10 µm.
